# Smooth muscle cell-specific deletion of TXNIP ameliorates medial vascular calcification

**DOI:** 10.1038/s12276-025-01474-5

**Published:** 2025-07-03

**Authors:** Ae-Rang Hwang, Chang-Hoon Woo

**Affiliations:** Department of Pharmacology and Senotherapy-Based Metabolic Disease Control Research Center, Yeungnam University College of Medicine, Daegu, Republic of Korea

**Keywords:** Calcification, Experimental models of disease

## Abstract

Vascular calcification is an important pathological characteristic of cardiovascular diseases, often exacerbated by metabolic disorders such as chronic kidney disease and diabetes mellitus. Recent studies have highlighted oxidative stress and inflammation as central mechanisms in the development of vascular calcification. While TXNIP is known to positively regulate reactive oxygen species generation and inflammasome activation, the specific contributions of TXNIP to vascular calcification have not been thoroughly elucidated. Here we aimed to elucidate the role of TXNIP in the pathogenesis of vascular calcification through combined in vitro and in vivo approaches. Medial calcification was evaluated by murine models of a 5/6 nephrectomy mouse model and a vitamin D3-induced mouse model with nephropathy. In vivo results demonstrate that heightened TXNIP expression in vascular smooth muscle cells correlates with increased calcification. This association is indicated by elevated calcium deposition, upregulation of osteogenic markers and enhanced mitochondria-derived reactive oxygen species production. By contrast, targeted genetic modifications to induce TXNIP deficiency in smooth muscle cells significantly mitigate these effects. Moreover, suppression of TXNIP in these models inhibited inflammasome activation, upregulation of mitotic regulators and mitochondrial dysfunction, suggesting a novel linkage between TXNIP activity and osteogenic differentiation pathways in medial calcification. The insights from this comprehensive study indicate that TXNIP not only amplifies oxidative stress and inflammation in vascular smooth muscle cells but also represents a potential therapeutic target for mitigating medial calcification. Modulating TXNIP expression or function may reduce the incidence of medial calcification in patients with cardiovascular diseases linked to metabolic disorders.

## TXNIP drives vascular calcification in cardiovascular diseases

Vascular calcification is a condition in which minerals build up in blood vessels, often due to imbalances in calcium and phosphate that can lead to heart disease, especially in people with chronic kidney disease. Researchers used mice to study the role of TXNIP in vascular calcification. They genetically modified mice to lack TXNIP in their vascular smooth muscle cells, which are cells that help blood vessels contract. They found that these modified mice had less calcification when exposed to high levels of vitamin D, which usually promotes calcification. The study showed that TXNIP contributes to calcification by increasing oxidative stress and inflammation in vascular smooth muscle cells. By reducing TXNIP, researchers observed improved mitochondrial function and less calcification. This suggests that targeting TXNIP could be a potential strategy for preventing vascular calcification in patients with chronic kidney disease or other related conditions.

This summary was initially drafted using artificial intelligence, then revised and fact-checked by the author.

## Introduction

Vascular calcification is a manifestation of cardiovascular disease (CVD) caused by abnormal deposition of minerals in the vascular system because of a phosphate–calcium imbalance. This condition can also arise from aging and diseases, including atherosclerosis, diabetes mellitus (DM) and chronic kidney disease (CKD)^1^. These factors contribute to increased morbidity and mortality rates associated with CVD, making them important risk factors for CVD-related mortality^2^. Moreover, they are robust predictors of vascular calcification.

Vascular smooth muscle cells (VSMCs) in the media layer are essential pluripotent vascular system cells with contractile phenotype. When VSMCs lose their contractile properties and change to a synthetic phenotype to cause vascular calcification, it can lead to reduced arterial elasticity, increased vascular stiffness, CVD, hypertension, coronary artery disease and CKD. In addition to aging and passive phosphorus accumulation or calcium deposition, recent studies point to this phenotypic transformation of VSMCs into osteoblast-like cells, as an essential step of vascular calcification and key mechanism of calcification^3–5^. CKD is one of the most common and general causes of vascular calcification^6^. Vascular calcification commonly occurs in patients receiving dialysis because of renal failure or impaired renal function^7^. Studies have reported that vascular calcification is found in CKD animal models^8^. In addition, other studies reported the association between impaired renal function and calcification to draw attention to the importance of such an association^9,10^.

It has been known that mitotic cell division is associated with the proliferation of VSMCs and that the abnormal activation of mitotic cell division causes vascular remodeling including atherosclerosis, hypertension and vascular calcification^11–14^. Mitotic overactivation or failure plays a crucial role in the progression of arterial stiffness and CVD; thus, new therapies targeting cell cycle regulation must be developed.

Among various factors that cause calcification, hyperphosphatemia is prevalent among patients with CKD and has been identified as a major cause of vascular calcification for a long time^15,16^. High phosphate levels induce not only the phenotypic transformation of VSMCs from contractile to synthetic but also the transformation to osteoblast-like cells, as well as differentiation into inflammatory cells^7,17^. Moreover, a series of mineral disorders and hormonal imbalances, including altered homeostasis of calcium, phosphate, vitamin D_3_ (Vit.D_3_; 1,25-dihydroxy vitamin D, calcitriol) and parathyroid hormone, cause a gradual decline in renal functions, leading to vascular calcification^18^. Calcitriol is produced in the kidneys as active vitamin D. Repetitive and abnormally excessive accumulation of Vit.D_3_ is known to cause kidney damage, and the resulting chronic loss of renal function makes Vit.D_3_ an appropriate drug for inducing calcification of the media layer. However, the primary mechanism by which this process happens in the CKD model has not been identified or completely understood.

Thioredoxin-interacting protein (TXNIP; also known as Vit.D_3_ upregulated protein 1 or thioredoxin-binding protein 2), which is expressed in various tissues and cells, binds directly to two active cysteines to interact with various intracellular proteins, including E3 ubiquitin ligase, NLR family pyrin domain containing 3 (NLRP3), and thioredoxin (Trx), to play a crucial role as a sensor in cellular signaling processes^19^. Moreover, TXNIP participates in biological and pathological processes such as inflammation, oxidative stress and apoptosis^20–24^. The primary role of TXNIP is pro-oxidative; it binds to and inhibits the antioxidative effects of Trx, thereby increasing reactive oxygen species (ROS) levels and inflammatory processes^25^. These mechanisms contribute to the development of CVD and cause permanent tissue damage. According to recent studies, TXNIP knockout (KO) can alleviate CKD processes, such as renal inflammation and renal fibrosis, by weakening oxidative stress, extracellular matrix accumulation, apoptosis and inflammatory processes associated with CVD^26,27^.

Regulation of the NLRP3 inflammasome signaling pathway can be a solution for effectively managing various diseases that appear as inflammation. TXNIP is upstream of NLRP3 and can activate the NLRP3 inflammasome by directly interacting with it^28^. A previous study reported that the binding of TXNIP to NLRP3 is essential for activating oxidative-stress-mediated NLRP3 inflammasomes in diabetic nephropathy^29^. High TXNIP expression associated with various pathological processes suggested that effective regulation of TXNIP expression can be a key mediator of various vascular diseases that appear as dysregulation of inflammation caused by oxidative stress. Reports indicate that TXNIP is essential in vascular remodeling and functions, in addition to its involvement in oxidative stress, inflammation and apoptosis^30^. In addition, activation of TXNIP in the CVD system induces hypertension, atherosclerosis, arterial stiffness and CKD^28,29^. By contrast, proinflammatory molecule expression and the cell-to-cell interaction between VSMCs and macrophages were inhibited in smooth muscle cell-specific TXNIP KO (TXNIP^∆SMC^) suggesting that TXNIP could be a potential target of CVD including vascular calcification^31^.

Given the close associations between oxidative stress, inflammation and VSMCs in CKD and various vascular inflammatory diseases, this study posits that genetic ablation of TXNIP expression in smooth muscle cells may inhibit vascular calcification. In light of TXNIP’s critical role in regulating oxidative stress and inflammation, our study aims to delineate the function of TXNIP in vascular calcification and explore its underlying molecular mechanisms.

## Materials and methods

### Animal studies

This study was conducted in accordance with the policies and regulations of the Institutional Animal Care and Use Committee of Yeungnam University College of Medicine (YUMC-AEC2023-010), and all animal procedures complied with the Directive 2010/63/EU of the European Parliament on the protection of animals used for scientific purposes. For the recovery animal experiments involving 5/6 nephrectomy, Zoletil (tiletamine and zolazepam) and xylazine (xylazine hydrochloride/tolazoline hydrochloride) were used as anesthetics. At the termination of the experiments, animals were euthanized using Avertin (tribromoethanol). Details on the use of anesthetics can be found in the section on 5/6 nephrectomy. *Txnip* floxed mice (#016847) and SM22α-CreKI mice (#006878) were used in the experiments, both purchased from The Jackson Laboratory. To generate smooth muscle cell-specific *Txnip*-deficient mice, we bred *Txnip* floxed mice with SM22α-CreKI mice. The mice were housed under a 12-h light–dark cycle, at a temperature of 22 °C and 40–50% humidity.

### VSMCs primary culture

For in vitro experiments, we isolated primary VSMCs from Sprague-Dawley rats (YUMC-AEC2023-010), TXNIP^WT^ and TXNIP^∆SMC^ mice (YUMC-AEC2023-002). Both rats and mice were anesthetized with intraperitoneal injections of a Zoletil (30 mg/kg) and xylazine (10 mg/kg) cocktail and Avertin (240 mg/kg). The isolation and culture of rat and mouse primary VSMCs were conducted as follows: the aortas were carefully excised, cleaned and washed with serum-free Dulbecco’s modified Eagle medium (DMEM; Welgene). The aortas were cut lengthwise, split in half and scraped with a sterile swab to remove endothelial cells. Afterward, the isolated aortas were chopped into approximately 1-mm pieces and cultured in DMEM with 50% fetal bovine serum (Welgene) and 1% antibiotics–antimycotics. VSMCs were identified using immunohistochemistry (IHC) with VSMC markers, either anti-α-SMA or anti-SM22α antibody, with more than 95% of total cells showing positivity. Primary cells used in this study were between passages 3 and 7.

### In vivo calcification mouse model

SM22α-specific *Txnip* transgenic mice were used in the present study. Six- to 8-week TXNIP wild-type (TXNIP WT; littermate control group) and TXNIP^∆SMC^ (TXNIP KO group) mice were used. For animal experiments, we randomly assigned mice to four groups: two TXNIP^WT^ groups and two TXNIP^∆SMC^ groups, and administered either the active form of vitamin D (Vit.D_3_; 500,000 IU per kilogram body weight per day) or 5% v/v ethanol subcutaneously for 3 days (*n* = 6–8 per group). The groups were TXNIP^WT^ control, TXNIP^WT^ + Vit.D_3_, TXNIP^∆SMC^ control, and TXNIP^∆SMC^ + Vit.D_3_. Six days after injection, we collected the aortas for pathological analysis and IHC, using Avertin (240 mg/kg, intraperitoneal injection (i.p.)) as an anesthetic. The aorta tissue was stored in 10% buffered formalin. The vitamin D solution was prepared using the following protocol: cholecalciferol (13.2 mg) was dissolved in a mixture of 40 µl of 100% ethanol and 280 µl of cremophor (Sigma-Aldrich), followed by incubation at room temperature for 5 min. This was then mixed with a solution of dextrose (150 mg dextrose dissolved in 3.68 µl of distilled water) and incubated again at room temperature for 5 min. The final solution was stored at 4 °C and prepared every day under the same protocol for 3 days for fresh use.

### 5/6 nephrectomy

The mice used in the 5/6 nephrectomy experiment were anesthetized with a single intraperitoneal injection of a mixture of Zoletil (tiletamine–zolazepam) at 40 mg/kg and xylazine (xylazine hydrochloride) at 5 mg/kg. The right kidney was 2/3 removed through an incision in the right flank. After 1 week, the left kidney was removed through an incision in the left flank, and 3–0 silk suture was used to tightly ligate the ureter. Subsequently, the left kidney was completely excised, and muscle and skin were sutured. After ligation, the animals were placed on top of a cage with a heat lamp (37 °C) and monitored until they regained consciousness. Analgesics and antibiotics were administered in 12-h intervals for 3 days to prevent infection and pain. The animals were provided a regular diet for 1 week after the surgery, followed by 12 weeks of diet containing a high dose of phosphorus (2%). At the termination of the experiments, animals were euthanized by initial anesthesia using Avertin (tribromoethanol), followed by cervical dislocation as a secondary method. A lethal dose of Avertin (250 mg/kg) was administered intraperitoneally to ensure rapid and humane euthanasia.

### In vitro calcification

Mouse or rat primary VSMC cells were seeded in a 6-well and 24-well culture plate at a density of 1 × 10^6^ and 0.7 × 10^5^ cells, respectively, grown to ~70–80% confluence and further incubated in the absence or presence of inorganic phosphate (Pi, 3 mM) or Vit.D_3_ (10^−7^ mol/l) for the indicated times.

### Statistical analysis

The data in bar graphs are presented as mean ± s.d., and the significance of intergroup differences was assessed by unpaired Student’s *t*-test and multiple group comparisons using analysis of variance (ANOVA) followed by Bonferroni’s post-hoc test, with *P* values <0.05 indicative of a significant difference. The analysis was conducted using GraphPad Prism (GraphPad Software).

## Results

### TXNIP expression increases in mouse models of medial calcification

**Fig. 1:**
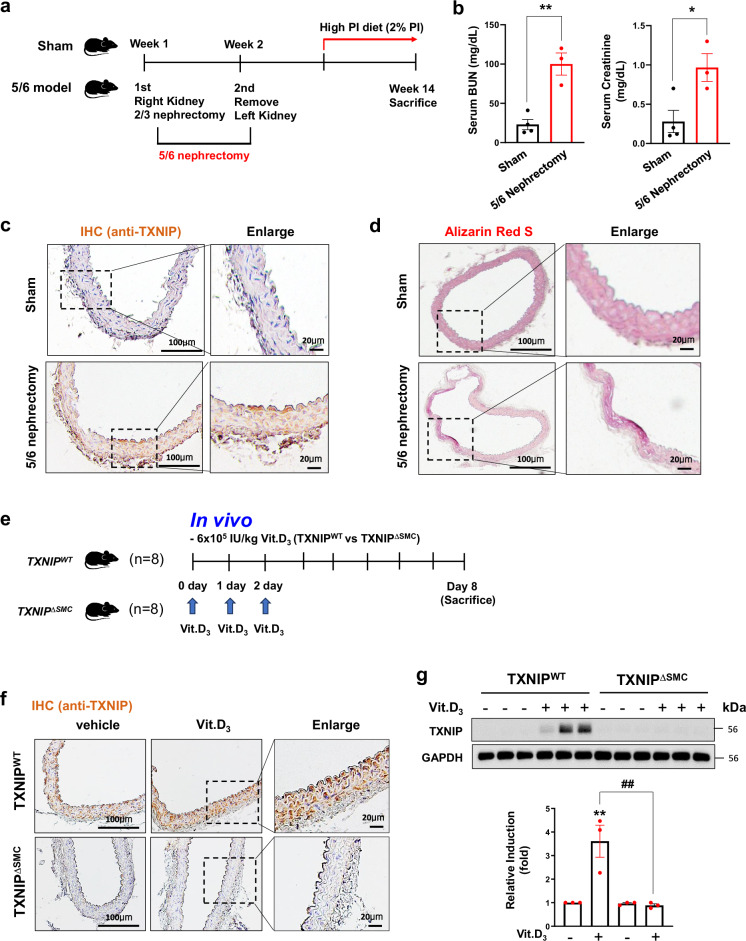
Increased TXNIP expression in murine models of vascular calcification. **a** A schematic diagram of the 5/6 nephrectomy animal model. **b** A comparison of plasma blood urea nitrogen (BUN) and creatinine levels in 5/6 nephrectomy animal model. *P < 0.05, **P < 0.01 versus vehicle (*n* = 4 per group). **c** TXNIP expression identified by IHC staining in 5/6 nephrectomized mice. Scale bars, 100 µm (left) and 20 µm (right) (*n* = 4 per group). **d** Calcification visualized by Alizarin Red S staining (positive staining: red). Scale bars, 100 µm (left) and 20 µm (right) (*n* = 4 per group). **e** A schematic diagram of the Vit.D_3_-related vascular calcification animal model. **f** IHC of TXNIP in the aortic VSMC layers of TXNIP^WT^ or TXNIP^∆SMC^ mice. The right panels are magnifications of the areas enclosed in squares in the central panels. Scale bars, 100 µm (left and middle) and 20 μm (right). TXNIP expression level was visualized using an optical microscope (*n* = 3 per group). **g** Western blot of TXNIP protein expression in isolated TXNIP^WT^ or TXNIP^∆SMC^ aortic VSMCs (*n* = 3 per group). GAPDH expression was used as a loading control. The bar graphs show the results of a densitometric analysis of immunoblot bands. Results are expressed as mean ± s.d. from three independent experiments. **P < 0.01 versus control, ^##^*P* < 0.01 versus TXNIP^WT^ Vit.D_3_ versus TXNIP^∆SMC^ + Vit.D_3._ The significance of differences between groups for all bar graph data was assessed using an unpaired Student’s *t*-test and ANOVA for multiple group comparisons.

We investigated the expression levels of TXNIP in vascular calcification using two murine models. First, a mouse model of 5/6 nephrectomy, which is a well-established model of CKD, was applied for medial calcification (Fig. 1a). As shown in Fig. 1b, creatinine and blood urea nitrogen levels were significantly increased in 5/6 nephrectomized mice fed with 2% Pi diet, suggesting impaired renal functions in 5/6 nephrectomized mice (Fig. 1b). In addition, immunohistochemical data revealed that protein expression of TXNIP was markedly increased in aortic smooth muscle layer in 5/6 nephrectomized mice compared with those of sham-operated mice (Fig. 1c). When Alizarin Red S staining, an indicator of the degree of calcification, was used to estimate the level of calcium deposition in the aorta of CKD mice, the results showed accumulation of calcium in aortic tissue of CKD mice (Fig. 1d). The findings showed that impaired renal function is associated with increased medial calcification and that TXNIP has a potential association in that process.

Second, to address the potential role of TXNIP in VSMCs during the development of vascular calcification, TXNIP^WT^ and TXNIP^∆SMC^ mice were applied for the murine model of Vit.D_3_-induced vascular calcification (Fig. 1e). The immunohistochemical data showed that TXNIP expression increased mostly in the smooth muscle layer of aorta from TXNIP^WT^ mice. In TXNIP^∆SMC^ mice, TXNIP signals were found partially in the endothelial and adventitia layers. However, there was no significant increase in TXNIP expression in VSMCs, suggesting smooth muscle cell-specific genetic depletion of TXNIP in TXNIP^∆SMC^ (Fig. 1f). Consistent with the IHC staining results, TXNIP protein expression in the aorta increased in TXNIP^WT^ mice, whereas the protein levels of TXNIP decreased in TXNIP^∆SMC^ mice (Fig. 1g). The increased expression of TXNIP, as observed, can similarly be confirmed in calcification-related diseases through analysis of human Gene Expression Omnibus (GEO) databases (GDS3712 and GDS3980). The TXNIP transcript level was significantly upregulated in humans with nephrosclerosis and DM (Supplementary Fig. 1a, b). These findings demonstrate the potential role of TXNIP in medial calcification.

### Smooth muscle cell-specific *Txnip* deficiency prevents Vit.D_3_-induced vascular calcification

**Fig. 2:**
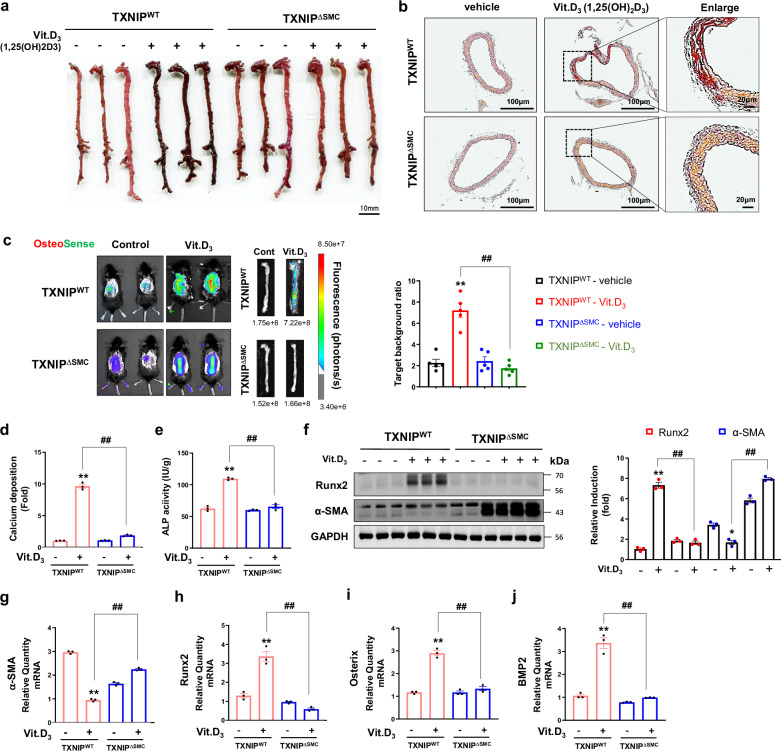
Roles of TXNIP in Vit.D_3_-induced vascular calcification in smooth muscle cell-specific TXNIP^∆SMC^ mice. **a**, **b**, Calcification of the whole aorta (**a**) and aortic tissue sections (**b**) was investigated by Alizarin Red S staining (positive staining: red) used to visualize calcium deposits. Scale bars, 10 mm (**a**), 100 µm (**b**, left and middle) and 20 µm (**b**, right) (*n* = 3 per group). **c** TXNIP^WT^ or TXNIP^∆SMC^ mice aorta was imaged after collection by the method described in the ‘Materials and methods’ section. At 24 h before euthanizing the animals, Osteosense 680 EX was injected intravenously, and the aorta was separated, after which fluorescence was measured and quantified graphically. Osteosense 680 EX dye binds to calcium at 675/720 nm to emit fluorescence, with darker-red fluorescence indicating greater calcium accumulation. Right: fluorescence intensity scale, photons/s. **P < 0.01 versus control, ^##^*P* < 0.01 versus TXNIP^WT^ + Vit.D_3_ versus TXNIP^∆SMC^ + Vit.D_3_ (*n* = 4 per group). **d**, **e** Aortic calcium content (**d**) in the serum of TXNIP^WT^ or TXNIP^∆SMC^ mice was determined by measurement of absorbance at 595 nm and quantified using a commercially available calcium detection kit. Serum ALP activity (**e**) was also determined by measurement of absorbance at 595 nm and quantified using a commercially available analysis kit. Data are expressed as mean ± s.d. from three independent experiments. **P < 0.01 versus control, ^##^*P* < 0.01 versus TXNIP^WT^ + Vit.D_3_ versus TXNIP^∆SMC^ + Vit.D_3_ (*n* = 3 per group). **f** Western blot of RUNX2 and α-SMA protein expression in isolated TXNIP^WT^ or TXNIP^∆SMC^ aortic VSMCs. GAPDH expression was used as a loading control. The bar graphs show the results of densitometric analysis of western blot bands. Data are expressed as mean ± s.d. from three independent experiments. *P < 0.05 or **P < 0.01 versus control, ^##^*P* < 0.01 versus TXNIP^WT^ + Vit.D_3_ versus TXNIP^∆SMC^ + Vit.D_3_ (*n* = 3 per group). **g**–**j** Effects of TXNIP^∆SMC^ on the mRNA level of calcification-related osteogenic genes (RUNX2 (**g**), osterix (**h**), BMP2 (**i**) and VSMC marker α-SMA (**j**)). The mRNA levels of calcification-related osteogenic genes and VSMC markers were determined by quantitative RT-PCR. Relative expression levels from samples were normalized by GAPDH. Data are representative of three independent experiments with similar results (shown as mean ± s.d.). **P < 0.01 versus control, ^##^*P* < 0.01 versus TXNIP^WT^ + Vit.D_3_ versus TXNIP^∆SMC^ + Vit.D_3_ (*n* = 3 per group). The significance of differences between groups for all bar graph data was assessed using an unpaired Student’s *t*-test and ANOVA for multiple group comparisons.

It has been known that the administration of high-dose Vit.D_3_ in human and animal models can cause toxicity, such as vascular calcification^32,33^. In addition, recent studies have elucidated the association between bone resorption and increased arterial calcification, as observed in Vit.D_3_-treated rats^16,20^. To investigate the functional role of TXNIP in vascular calcification in vivo, high-dose Vit.D_3_, an active form of vitamin D, was administered to TXNIP^WT^ and TXNIP^∆SMC^ mice. Alizarin Red S staining revealed that extensive calcification was induced mainly in the VSMC layer of the whole aorta and aorta tissue sections from TXNIP^WT^, whereas Vit.D_3_-mediated calcification in the aorta was reduced in TXNIP^∆SMC^ mice (Fig. 2a, b). For a precise evaluation of vascular calcification, Osteosense dye was intravenously injected 24 h before collecting tissue from TXNIP^WT^ and TXNIP^∆SMC^ mice that received three consecutive days of Vit.D_3_ injection, and the level of fluorescence was measured using a Spectral Ami/Ami X machine. A distinct increase of in vivo calcium deposition in the aorta of TXNIP^WT^ mice that received Vit.D_3_ injection was detected, whereas TXNIP^∆SMC^ mice showed a significant decrease in calcium deposition in the aorta (Fig. 2c). Moreover, calcium accumulation and alkaline phosphatase (ALP) activity were significantly increased in aorta from Vit.D_3_-treated TXNIP^WT^ mice compared with the vehicle control, and these inductions were significantly reduced in TXNIP^∆SMC^ mice, indicating that TXNIP in VSMC is a positive regulator for the development of vascular calcification (Fig. 2d, e). For addressing the mechanism of TXNIP-mediated vascular calcification, molecular markers for the calcification process were measured. Runx2 is a marker that increases in the calcification process, and α-SMA is a representative marker that decreases during the same process. In this study, Runx2 and α-SMA were used as major markers to evaluate osteogenic differentiation. Western blots and quantitative real time reverse transcription polymerase chain reaction (qRT-PCR) results showed increased protein and mRNA levels of calcification-related molecules (Runx2, Osterix and BMP2) and decreased levels of a VSMC marker (α-SMA) in aorta tissue from TXNIP^WT^ mice, but not in aortic tissue from TXNIP^∆SMC^ mice (Fig. 2f, g–j). These results show that TXNIP^∆SMC^ prevents vascular calcification by inhibiting cell differentiation mediated by Vit.D_3_-induced phenotype transformation of VSMCs into osteoblast-like cells.

### Depletion of TXNIP reduced calcification in primary VSMCs

**Fig. 3:**
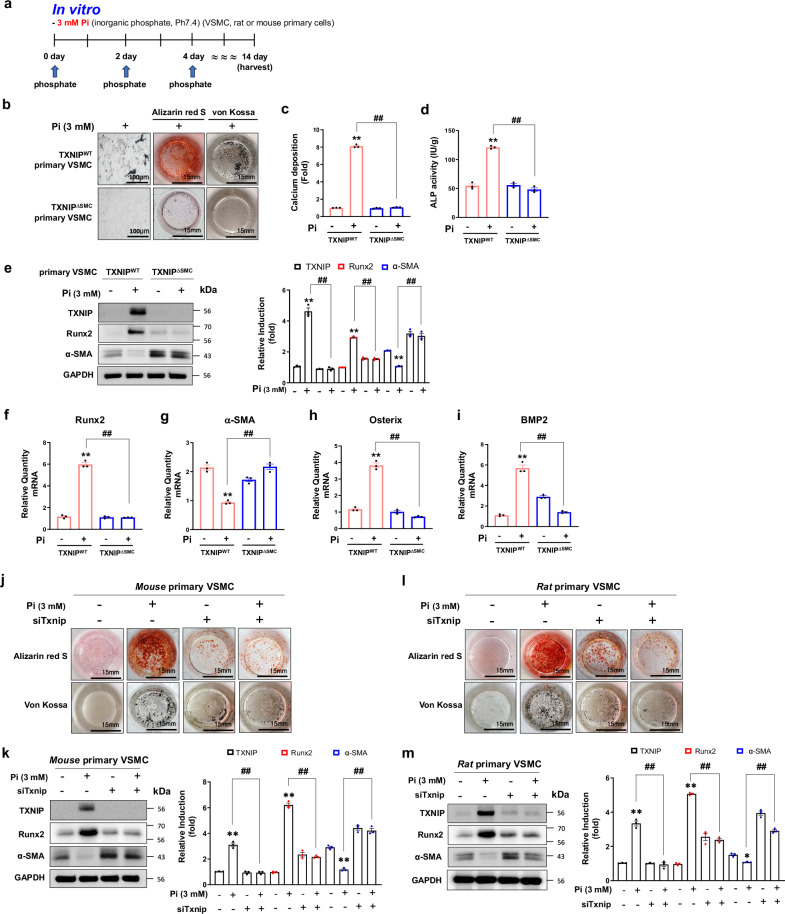
Gene silencing of TXNIP prevents Pi-induced calcification in rat and mouse VSMCs. **a** A schematic diagram of the study plan for inducing calcification in VSMCs isolated from TXNIP^WT^ and TXNIP^∆SMC^ mice. **b** Calcification was visualized by Alizarin Red S (positive staining: red) or von Kossa (positive staining: black) staining. The primary VSMCs isolated from TXNIP^WT^ and TXNIP^∆SMC^ mice were cultured for 14 days in an osteogenic medium containing a high dose of phosphate. Scale bar, 100 µm; well diameter, 15 mm (*n* = 3 per group). **c**, **d** Calcium accumulation (**c**, 595 nm) and ALP activity (**d**, 450 nm) induced by exposure to a high dose of phosphate in primary VSMCs isolated from TXNIP^WT^ or TXNIP^∆SMC^ mice were measured by the absorbance at respectively wavelength and quantified graphically (*n* = 3 per group). Data are representative of three independent experiments with similar results (shown as mean ± s.d.). **P < 0.01 versus control, ^##^*P* < 0.01 versus TXNIP^WT^ + Pi versus TXNIP^∆SMC^ + Pi (*n* = 3). **e** The protein expression of TXNIP, Runx2 and VSMC contractile gene marker (α-SMA) in primary smooth muscle cells isolated from TXNIP^WT^ and TXNIP^∆SMC^ mice is shown as immunoblot results. GAPDH expression was used as a loading control. The bar graphs show the results of densitometric analysis of immunoblot bands. Results are expressed as mean ± s.d. from three independent experiments. **P < 0.01 versus control, ^##^*P* < 0.01 versus TXNIP^WT^ + Pi versus TXNIP^∆SMC^ + Pi (*n* = 3 per group). **f**–**i** Analysis of the mRNA levels of calcification-related osteogenic genes RUNX2 (**f**), osterix (**g**), BMP2 (**h**) and VSMC contractile gene marker α-SMA (**i**) by RT-PCR. Results are expressed as mean ± s.d. from three independent experiments. **P < 0.01 versus control, ^##^*P* < 0.01 versus TXNIP^WT^ + Pi versus TXNIP^∆SMC^ + Pi (*n* = 3 per group). Primary VSMCs isolated from Sprague-Dawley rats or TXNIP^WT^ mice were transfected for 48 h with negative control siRNA or Txnip siRNA (siTxnip). Subsequently, the cells were cultured in a medium containing DMEM or high-dose phosphate (3 mM) replaced in 2-day intervals. On day 8, the final degree of calcification was analyzed. **j**, **l** Calcium accumulation was identified using Alizarin Red S (positive staining: red) or von Kossa staining (positive staining: black). Well diameter, 15 mm (*n* = 3 per group). **k**, **m** Protein expression of TXNIP, Runx2 and VSMC contractile gene marker α-SMA was identified by immunoblotting in primary VSMCs isolated from mouse (**k**) and rat (**m**). Protein expression band densitometry results are presented as bar graphs. GAPDH expression was used as a loading control. Results are expressed as mean ± s.d. from three independent experiments. *P < 0.05 or **P < 0.01 versus control, ^##^*P* < 0.01 versus Pi versus siTxnip + Pi (*n* = 3 per group). The significance of differences between groups for all bar graph data was assessed using an unpaired Student’s *t*-test and ANOVA for multiple group comparisons.

To investigate the importance of TXNIP in calcifying VSMCs, primary VSMCs were obtained from TXNIP^WT^ and TXNIP^∆SMC^ mice and the high-dose Pi-treated calcification in vitro model was investigated (Fig. 3a). As determined by Alizarin Red S and von Kossa stainings for visual confirmation of the degree of calcium accumulation, the primary VSMCs of TXNIP^WT^ showed a significant increase in the degree of calcium accumulation after exposure to Pi, whereas the primary VSMCs of TXNIP^∆SMC^ mice showed reduced calcifying phenotypes (Fig. 3b). In addition, high-dose Pi-induced calcium accumulation and ALP activity were significantly inhibited in the primary VSMCs of TXNIP^∆SMC^ mice compared with those of TXNIP^WT^ mice (Fig. 3c, d). We also found that Pi-induced molecular marker expressions were reversed in primary VSMCs of TXNIP^∆SMC^ (Fig. 3e–i). These findings indicate that TXNIP exhibits the ability to promote the calcification and transformation to an osteogenic phenotype in VSMCs.

In addition to genetic depletion of TXNIP, to examine whether transient knockdown of TXNIP has similar effects on vascular calcification, a small interfering RNA (siRNA) system was used to inhibit the *Txnip* gene in mouse and rat primary VSMCs. High-dose Pi-induced calcification was significantly inhibited in mouse primary VSMCs transfected with Txnip siRNA (Fig. 3j, k). We also found a similar pattern in rat primary VSMCs with Txnip siRNA (Fig. 3l, m). These results suggest that depletion of TXNIP inhibited the development of calcification by preventing VSMC phenotype change to osteogenic phenotype. High-dose Vit.D_3_-induced calcification was also significantly inhibited in primary VSMCs from TXNIP WT mice, and these phenotypic changes were reversed in primary VSMCs from TXNIP^∆SMC^ mice (Supplementary Fig. 2a, c). In addition, high-dose Vit.D_3_-induced molecular marker expressions were reversed in primary VSMCs of TXNIP^∆SMC^ (Supplementary Fig. 2d–h). These results suggest that TXNIP^∆SMC^ could prevent calcifying VSMCs induced by Pi as well as Vit.D_3_.

### TXNIP-dependent NLRP3 inflammasome activity is involved in vascular calcification

**Fig. 4:**
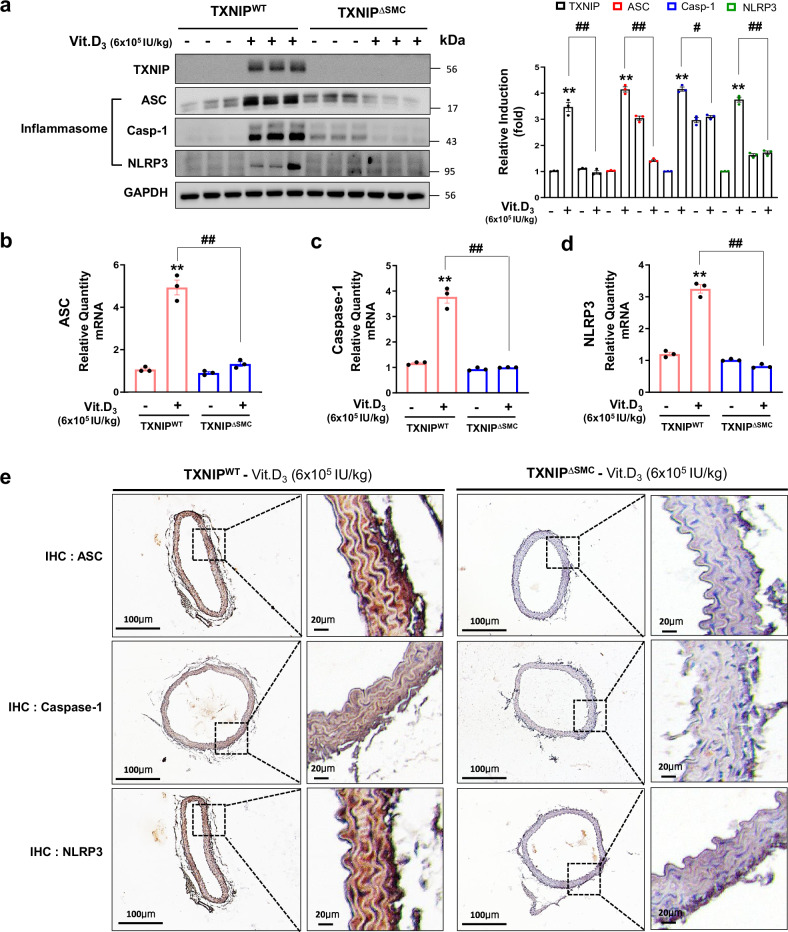
TXNIP promotes high-dose Vit.D_3_-induced NLRP3 inflammasome formation in mouse aorta. **a** Relative mRNA expression of NLRP3 inflammasome components (ASC (**b**), Caspase-1 (**c**), and NLRP3 (**d**)) in the aorta of TXNIP mice with calcification induced by high-dose Vit.D_3_ injection were analyzed by immunoblotting. Protein expression band densitometry results are given as bar graphs. GAPDH expression was used as a loading control. Results are expressed as mean ± s.d. from three independent experiments. **P < 0.01 versus control, ^#^*P* < 0.05, ^##^*P* < 0.01 versus TXNIP^WT^ + Vit.D_3_ versus TXNIP^∆SMC^ + Vit.D_3_ (*n* = 3 per group). **b**–**d** Relative mRNA expression of NLRP3 inflammasome components in the aorta of the vehicle or Vit.D_3_-treated TXNIP^WT^ or TXNIP^∆SMC^ mice. The molecular mechanisms involved in the calcification process were analyzed by qPCR. Results are expressed as mean ± s.d. from three independent experiments. **P < 0.01 versus control, ^##^*P* < 0.01 versus TXNIP^WT^ + Vit.D_3_ versus TXNIP^∆SMC^ + Vit.D_3_ (*n* = 3 per group). **e** The expression of NLRP3 inflammasome components (ASC, Casp-1 and NLRP3) in VSMCs was assessed by IHC staining (positive staining: brown). Scale bars, 100 μm (left) and 20 μm (right). The right panels are magnifications of the areas enclosed in squares in the left panels (*n* = 3 per group). ASC, Casp-1 and NLRP3 IHC expression levels were visualized using an optical microscope. The significance of differences between groups for all bar graph data was assessed using an unpaired Student’s *t*-test and ANOVA for multiple group comparisons.

It has been reported that VSMC calcification was prevented by inhibiting NLRP3 inflammasome activity, indicating that inhibiting NLRP3 activity may have an important role in the development of vascular calcification^34,35^. Studies have reported that TXNIP is considered the primary target interacting with NLRP3 of the NLRP3-dependent inflammasome complex^26^. To investigate whether TXNIP promotes vascular calcification by mediating inflammatory response, an in vivo study analyzed the expression of NLRP3 inflammasome-mediated inflammatory mediators caused by Vit.D_3_ in the aorta of TXNIP^WT^ and TXNIP^∆SMC^ mice. The western blot results showed distinct increases in the expression of TXNIP and NLRP3 inflammasome components (ASC, Casp-1 and NLRP3) in the aorta of TXNIP^WT^ mice, whereas they showed a decrease in protein expression of NLRP3 inflammasome-related markers in the aorta of TXNIP^∆SMC^ mice (Fig. 4a). Similar results were obtained with IHC in the aorta (Fig. 4e). In addition to protein expression, the mRNA level of NLRP3 inflammasome components (ASC, Casp-1 and NLRP3) increased significantly after the administration of a high dose of Vit.D_3_ compared with that in the TXNIP^WT^ control. On the contrary, the increased expression of inflammasome components was reversed in TXNIP^∆SMC^ mice (Fig. 4b–d). These results suggest that the TXNIP-dependent NLRP3 inflammasome activation could be involved in the development of vascular calcification.

### TXNIP-dependent oxidative stress in a mouse model of Vit.D_3_-induced vascular calcification

**Fig. 5:**
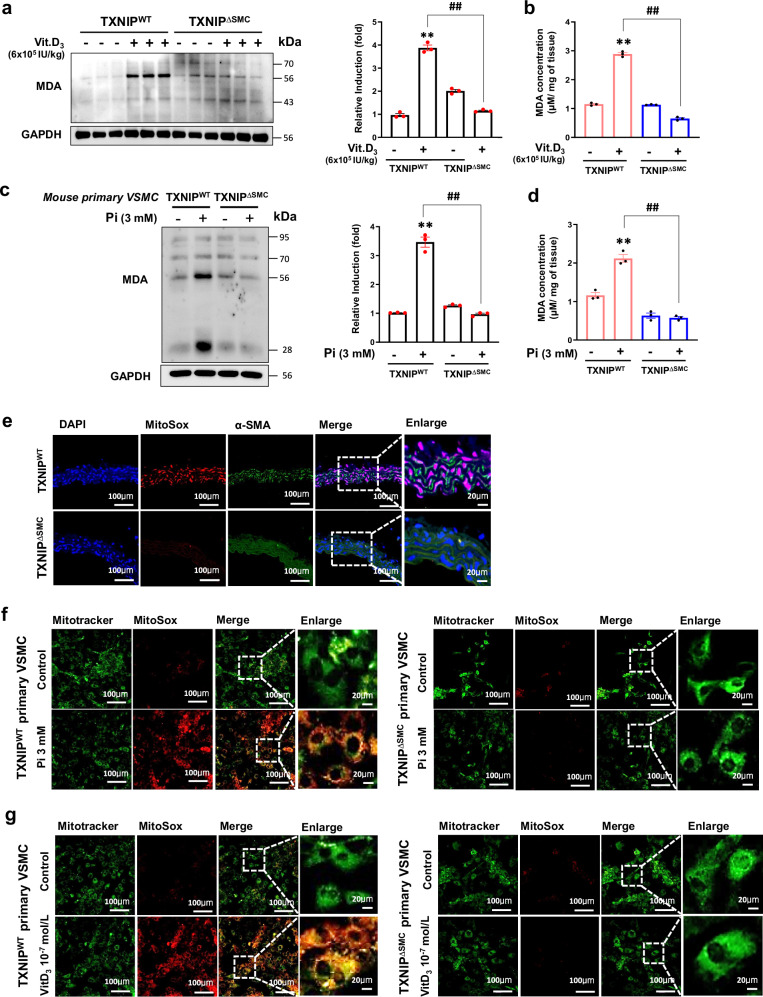
TXNIP promotes high-dose Vit.D_3_- and Pi-induced oxidative stress in mouse aorta and primary VSMCs, respectively. In the in vivo studies, calcification models were established by subcutaneous injecting a high dose of Vit.D_3_ (6 × 10^5^ IU/kg) for three consecutive days (daily dose of 100 μl per 25 g) to 8-week-old TXNIP^WT^ and TXNIP^∆SMC^ mice. In the in vitro studies, primary VSMCs isolated from TXNIP^WT^ and TXNIP^∆SMC^ mice were cultured for 14 days in an osteogenic medium containing high-dose Pi and Vit.D_3_. **a** MDA protein expression in the aorta isolated from TXNIP^WT^ and TXNIP^∆SMC^ mice after injection of high-dose Vit.D_3_ for three consecutive days, identified by immunoblotting. GAPDH expression was used as a loading control. Protein expression band densitometry results are given as bar graphs. **P < 0.01 versus control, ^##^*P* < 0.01 versus TXNIP^WT^ + Vit.D_3_ versus TXNIP^∆SMC^ + Vit.D_3_ (*n* = 3 per group). **b** Lipid peroxidation in the aortic tissue samples of TXNIP^WT^ and TXNIP^∆SMC^ mice was analyzed using an MDA concentration assay kit and measured by enzyme-linked immunosorbent assay (ELISA; 530—540 nm). Results are expressed as mean ± s.d. from three independent experiments. **P < 0.01 versus control, ^##^*P* < 0.01 versus TXNIP^WT^ + Vit.D_3_ versus TXNIP^∆SMC^ + Vit.D_3_ (*n* = 3 per group). **c** MDA concentration in primary VSMCs isolated from TXNIP^WT^ and TXNIP^∆SMC^ mice. MDA protein expression in cells was analyzed by immunoblotting. GAPDH expression was used as a loading control. Protein expression band densitometry results are given as bar graphs. Results are expressed as mean ± s.d. from three independent experiments. **P < 0.01 versus control, ^##^*P* < 0.01 versus TXNIP^WT^ + Vit.D_3_ versus TXNIP^∆SMC^ + Vit.D_3_ (*n* = 3 per group). **d** Lipid peroxidation in mouse primary VSMC lysate was measured by ELISA. Results are expressed as mean ± s.d. from three independent experiments. **P < 0.01 versus control, ^##^*P* < 0.01 versus TXNIP^WT^ + Vit.D_3_ versus TXNIP^∆SMC^ + Vit.D_3_ (*n* = 3 per group). **e** MitoSox (red) expression level in the calcified aorta of TXNIP^WT^ and TXNIP^∆SMC^ mice identified by in vivo immunofluorescence staining. Aorta tissue was prepared as 5-μm frozen sections after inducing calcification. The nucleus and aortic VSMCs were stained by 4′,6-diamidino-2-phenylindole (DAPI, blue) and α-SMA (green), respectively. Combined images are shown. Scale bars, 100 µm and 20 µm (enlarged) (*n* = 5 per group). **f**, **g** Mitochondrial MitoSox (red) expression in primary VSMCs of TXNIP^WT^ and TXNIP^∆SMC^ mice with calcification induced by the treatment of high-dose Pi (**f**) and Vit.D_3_ (**g**) was identified by immunofluorescence staining. Mitochondria are marked by MitoTracker (green). Combined images are shown. Scale bars, 100 µm and 20 µm (enlarged) (*n* = 5 per group). The significance of differences between groups for all bar graph data was assessed using an unpaired Student’s *t*-test and ANOVA for multiple group comparisons.

Inflammatory responses increase ROS levels, leading to functional impairments by damaging endothelial cells and VSMCs that cause various vascular pathologies including calcification^36^. Owing to the pivotal role of TXNIP in mediating oxidative stress, this study evaluated the potential involvement of TXNIP in vascular calcification mediated by ROS generation in VSMCs. Figure 5a shows the in vivo study results demonstrating the levels of ROS in the aorta of TXNIP^WT^ and TXNIP^∆SMC^ mice, assessed using malondialdehyde (MDA), a well-established marker of ROS-dependent lipid peroxidation. Western blot results showed a distinct increase in MDA protein expression in the aorta of TXNIP^WT^ mice, whereas TXNIP^∆SMC^ mice showed a reduced level of MDA (Fig. 5a). In addition, lipid peroxidation levels were measured to assess the content of MDA in the aorta. The exposure to Vit.D_3_ caused an increase in lipid peroxidation content in tissues different from that in TXNIP^WT^ control mice; this increase was reversed in TXNIP^∆SMC^ mice (Fig. 5b). In vitro studies using primary VSMCs isolated from mice and rats showed a similar pattern of results. Pi-induced MDA expression levels were significantly reduced in VSMCs of TXNIP^∆SMC^ mice compared with those of TXNIP^WT^ mice, suggesting a role of TXNIP-dependent oxidative stress in vascular calcification (Fig. 5c, d).

The mitochondria are essential for energy metabolism and maintain the balance between ROS generation and antioxidant effects. However, various pathological conditions, including calcification of VSMCs, disrupt the balance between oxidative and antioxidant effects, causing mitochondria-derived oxidative stress that leads to changes in VSMCs to an osteogenic phenotype^37,38^. Therefore, to investigate the role of mitochondria as the source of ROS increased by calcification inducer, MitoSox–MitoTracker staining, an indicator that can identify mitochondria-derived oxidative stress, was used to visualize the level of mitochondria-derived ROS in the aortic tissue and living cells. In the in vivo study, the level of mitochondria-derived ROS in the aorta of TXNIP^WT^ mice that received an administration of high-dose Vit.D_3_ showed increased oxidative stress in the media layer, whereas mitochondrial ROS was reduced in the aorta of TXNIP^∆SMC^ mice (Fig. 5e). In vitro, mitochondria-derived ROS in VSMCs of TXNIP^WT^ and TXNIP^∆SMC^ mice was investigated. When calcification was induced by treatment with two calcification inducers, Pi (3 mM) and Vit.D_3_ (10^−7^ mol/l), mitochondria-derived ROS was significantly higher in the VSMCs of TXNIP^WT^ mice, as compared with that in the cells of TXNIP^∆SMC^ mice and the control, whereas the VSMCs of TXNIP^∆SMC^ mice showed significantly reduced mitochondria ROS expression (Fig. 5f, g). These findings suggest that TXNIP is a key regulator in mitochondrial ROS generation for vascular calcification.

### *Txnip* deficiency improves mitochondrial function and inhibits mitochondrial oxidative stress

**Fig. 6:**
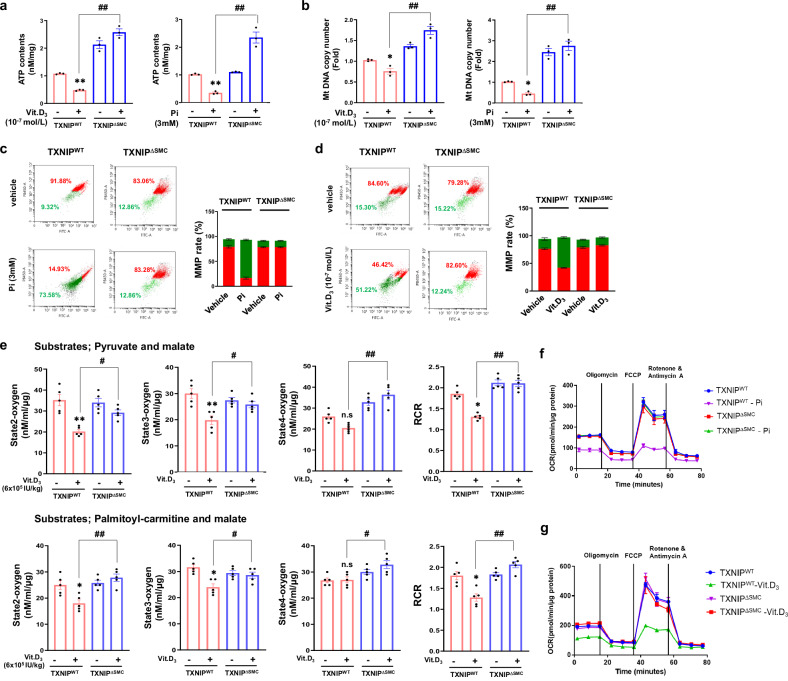
*Txnip* deficiency prevents mitochondrial dysfunction in TXNIP^∆SMC^ mice. **a** Mitochondria-derived ATP production was analyzed using an ATP detection assay kit. Results are expressed as mean ± s.d. from three independent experiments. **P < 0.01 versus control, ^#^*P* < 0.05 versus TXNIP^WT^ + Vit.D_3_ versus TXNIP^∆SMC^ + Vit.D_3,_^##^*P* < 0.01 versus TXNIP^WT^ + Pi versus TXNIP^∆SMC^ + Pi (*n* = 3 per group). **b** Analysis of mtDNA copy number in VSMCs of TXNIP^WT^ mice with calcification induced by high-dose Vit.D_3_. The copy number of mtDNA was determined by quantitative RT-PCR. Results are expressed as mean ± s.d. from three independent experiments. *P < 0.05 versus control, ^##^*P* < 0.01 versus TXNIP^WT^ + Vit.D_3_ or Pi versus TXNIP^∆SMC^ + Vit.D_3_ or Pi (*n* = 3 per group). **c**, **d** JC-1 staining for monitoring MMP in VSMCs of TXNIP^WT^ mice with calcification induced by high-dose Pi (**c**) and Vit.D_3_ (**d**) (red: JC-1 aggregates, green: JC-1 monomers). Mitochondrial depolarization is expressed as a ratio of aggregates to monomers. JC-1 staining results on the mean fluorescence intensity ratio of aggregates to monomers are quantified graphically (*n* = 3). MMP identified by JC-1 staining was determined by fluorescence-activated cell sorting (FACS) analysis (*n* = 3 per group). **e** Measurement of mitochondria oxygen consumption in the aorta with calcification induced by injection of high-dose Vit.D_3_. States 2, 3 and 4 of respiration and RCR with pyruvate, palmitoyl-carnitine and malate as substrates. Results are expressed as mean ± s.d. from three independent experiments. *P < 0.05, **P < 0.01 versus control, ^#^*P* < 0.05, ^##^*P* < 0.01 versus TXNIP^WT^ + Vit.D_3_ versus TXNIP^∆SMC^ + Vit.D_3_ (*n* = 5 per group). **f**, **g** Oxygen consumption rates in VSMCs treated with high-dose Pi (**f**) and Vit.D_3_ (**g**) as measured with a Seahorse XF24 analyzer. Oligomycin (1.5 μM, an inhibitor of complex V), FCCP (1.0 μM, an uncoupling agent that promotes transport of hydrogen ions), rotenone (0.5 μM, an inhibitor of complex I) and antimycin A (0.5 μM, an inhibitor of complex III) were added at the indicated points (*n* = 3 per group). The significance of differences between groups for all bar graph data was assessed using an unpaired Student’s *t*-test and ANOVA for multiple group comparisons.

Damage to mitochondrial function promotes mitochondria-derived oxidative stress, which is critical for calcification of VSMCs^39–41^. Thus, we aimed to elucidate the mechanism underlying the TXNIP-induced vascular calcification attributable to mitochondrial dysfunction. First, we found that Vit.D_3_ and Pi reduced mitochondria-derived ATP production in VSMCs of TXNIP^WT^ mice, whereas VSMCs of TXNIP^∆SMC^ facilitated ATP production (Fig. 6a). In addition, we observed a similar pattern with mitochondria DNA copy number, suggesting that TXNIP reduces mitochondrial function in calcifying VSMCs (Fig. 6b).

Next, to investigate the role of TXNIP on mitochondrial function in calcified VSMCs, mitochondrial membrane potential (Δ*Ψ*_m_, MMP), another indicator that can be used to identify mitochondrial function, was analyzed. JC-1 staining was used to identify MMP by analyzing mitochondrial outer membrane permeability. VSMCs of TXNIP^WT^ mice cultured in an osteogenic medium containing Pi and Vit.D_3_ showed decreased MMP in calcified VSMCs, as compared with that of control VSMCs, whereas TXNIP^∆SMC^ VSMCs showed restored MMP (Fig. 6c, d).

For a more detailed investigation of the role of TXNIP in modulating mitochondrial function, mitochondrial oxygen consumption was investigated. Mitochondrial oxygen consumption was measured in the aortic tissue of TXNIP^WT^ and TXNIP^∆SMC^ mice with calcification induced by high-dose Vit.D_3_. When pyruvate and malate were used as the substrate, TXNIP^∆SMC^ mice showed significantly restored mitochondrial state 2 (substrate-stimulated oxygen consumption), state 3 respiration (ADP-dependent oxygen consumption) and state 4 respiration (oxygen consumption in the absence of ATP synthesis) in aortic VSMCs compared with those of TXNIP^WT^ mice. Similar results were observed when palmitoyl-carmitine and malate were used as substrates, meaning TXNIP^∆SMC^ increased respiratory control ratio (RCR) regardless of the type of substrate (Fig. 6e). Such findings demonstrated that TXNIP^∆SMC^ improved mitochondrial function and respiration in a calcified state. Mitochondrial respiratory capacity was analyzed using Oxytherm System. Furthermore, we performed the Seahorse XF Cell Mito Stress Test to investigate the effects of TXNIP on the mitochondrial functions of VSMCs in vitro. We observed the effects of mitochondrial inhibitors oligomycin, FCCP and rotenone–antimycin A over time. As shown in Fig. 6f, g, mitochondrial basal OCR was substantially reduced in primary VSMCs from TXNIP^WT^ mice induced by high concentrations of Pi and Vit.D_3_, but not in those from TXNIP^∆SMC^ mice. These findings suggest that TXNIP^∆SMC^ can limit or reverse vascular calcification by improving mitochondrial function and respiration and inhibiting mitochondria-derived oxidative stress in calcified aorta and primary VSMCs.

### TXNIP overexpression promotes mitochondrial dysfunction and oxidative stress

**Fig. 7:**
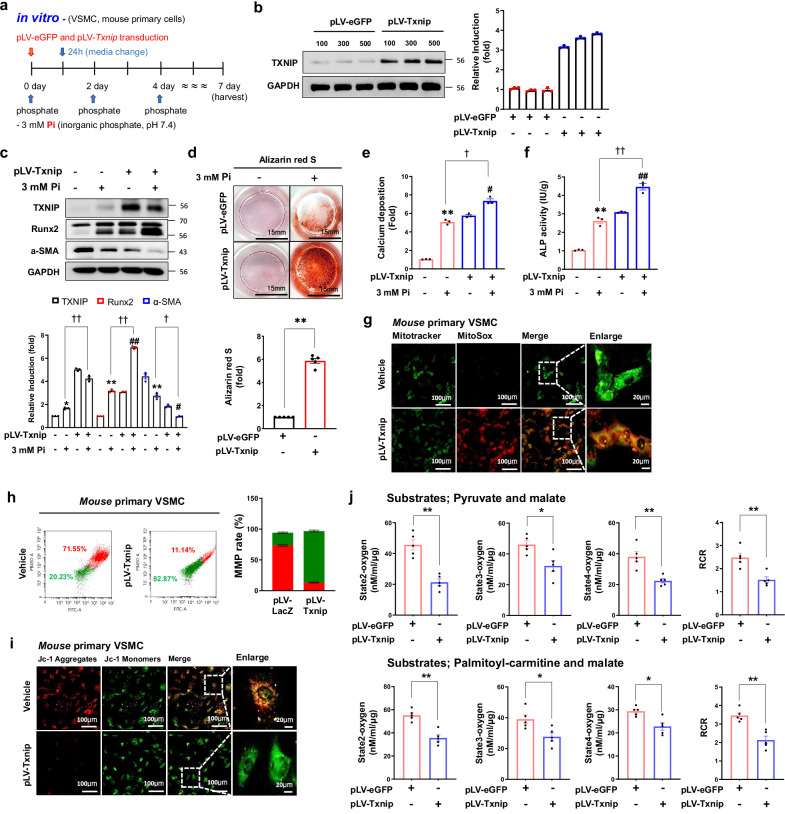
TXNIP overexpression induces calcification and mitochondrial dysfunction in primary VSMCs. **a** A schematic of calcification in primary VSMCs induced by TXNIP overexpression. **b** TXNIP protein expression in primary VSMCs mice transduced for 48 h with TXNIP overexpression lentivirus (pLV–Txnip) or empty lentivirus (pLV–eGFP). GAPDH expression was used as a loading control. Protein expression band densitometry results are given as bar graphs. Results are expressed as mean ± s.d. from three independent experiments (*n* = 3 per group). **c** After transducing primary VSMCs with pLV–Txnip for 48 h, the cells were cultured in an osteogenic medium containing high-dose phosphate for 7 days. Measurement of protein expression of calcification-related gene (Runx2) and VSMC contractile gene marker (α-SMA). Protein expression of TXNIP, Runx2 and α-SMA was analyzed by western blotting. GAPDH expression was used as a loading control. Protein expression band densitometry results are given as bar graphs. Results are expressed as mean ± s.d. from three independent experiments. *P < 0.05, **P < 0.01 versus control, ^#^*P* < 0.05, ^##^*P* < 0.01 versus pLV–Txnip, ^†^*P* < 0.05, ^††^*P* < 0.01 versus Pi versus pLV–Txnip + Pi (*n* = 3 per group). **d** Micro-calcification in primary VSMCs was determined by Alizarin Red S staining. The graphs show the quantification of Alizarin Red S positive staining. *P < 0.05 versus control. Well diameter, 15 mm (*n* = 3 per group). **e**, **f** Calcium content (**e**) and ALP activity (**f**) in primary VSMCs after high-dose Pi injection and pLV–Txnip transduction were analyzed by ELISA. Results are expressed as mean ± s.d. from three independent experiments. **P < 0.01 versus control, ^#^*P* < 0.05, ^##^*P* < 0.01 versus pLV–Txnip, ^†^*P* < 0.05, ^††^*P* < 0.01 versus Pi versus pLV–Txnip + Pi (*n* = 3 per group). **g** Measurement of mitochondria-derived superoxide production by MitoSox (red) staining in calcified primary VSMCs transduced with pLV–Txnip. Scale bars, 100 µm and 20 µm (enlarged) (*n* = 5 per group). **h** JC-1 staining for monitoring MMP in primary mouse VSMCs with calcification induced by TXNIP overexpression (red: JC-1 aggregates, green: JC-1 monomers). JC-1 staining results on the mean fluorescence intensity ratio of aggregates to monomers are quantified graphically (*n* = 3 per group). MMP was determined by FACS analysis. Results are expressed as mean ± s.d. from three independent experiments. **i** MMP in VSMCs with calcification induced by pLV–Txnip transduction analyzed by JC-1 dye staining (red: JC-1 aggregates, green: JC-1 monomers). Scale bars, 100 µm and 20 µm (enlarged). The ratio of JC-1 red/green fluorescence intensity was graphically analyzed. Results are expressed as mean ± s.d. from three independent experiments. **P < 0.01 versus control (*n* = 5 per group). **j** Measurement of mitochondrial oxygen consumption in VSMCs with calcification induced by TXNIP overexpression using Oxytherm System. States 2, 3 and 4 of respiration and RCR with pyruvate, palmitoyl-carmitine and malate as substrates. Results are expressed as mean ± s.d. from three independent experiments. *P < 0.05, **P < 0.01 versus control (*n* = 5 per group). The significance of differences between groups for all bar graph data was assessed using an unpaired Student’s *t*-test and ANOVA for multiple group comparisons.

To examine whether TXNIP itself participates in progression of calcifying VSMCs, TXNIP overexpression using the lentiviral system was applied under Pi induction (Fig. 7a). Western blot analysis showed that lentiviral transduction increased TXNIP expression in a dose-dependent manner (Fig. 7b). More importantly, TXNIP overexpression in primary VSMCs showed increased TXNIP and calcification-related marker (Runx2) expression, as well as decreased expression of contractile VSMC phenotype marker (α-SMA) (Fig. 7c). We also confirmed that TXNIP overexpression increased calcium deposition and ALP activity (Fig. 7d–f).

Next, whether TXNIP overexpression can cause mitochondria-derived oxidative stress was investigated by MitoSox–MitoTracker immunofluorescence staining. The results showed that mitochondria-derived oxidative stress significantly increased in VSMCs transduced with lentivirus encoding TXNIP compared with VSMCs transduced with lentivirus encoding LacZ (Fig. 7g). This study confirmed that TXNIP overexpression promotes not only mitochondria-derived oxidative stress but also calcification. Whether TXNIP can regulate mitochondrial function was investigated by identifying the MMP levels caused by TXNIP overexpression using JC-1 staining. Flow cytometer and JC-1 immunofluorescence staining results showed that TXNIP overexpression reduced the MMP in VSMCs (Fig. 7h, i). In addition, we found that TXNIP overexpression promoted decreases in mitochondrial OCR, suggesting that TXNIP promotes mitochondrial dysfunction through reducing MMP and OCR in VSMCs (Fig. 7j). These findings suggest that TXNIP causes oxidative stress to transform VSMCs into an osteoblast-like phenotype, further accelerating the calcification process via mitochondrial dysfunction.

### TXNIP overexpression results in alterations of genes related to the mitotic cell cycle in VSMCs

**Fig. 8:**
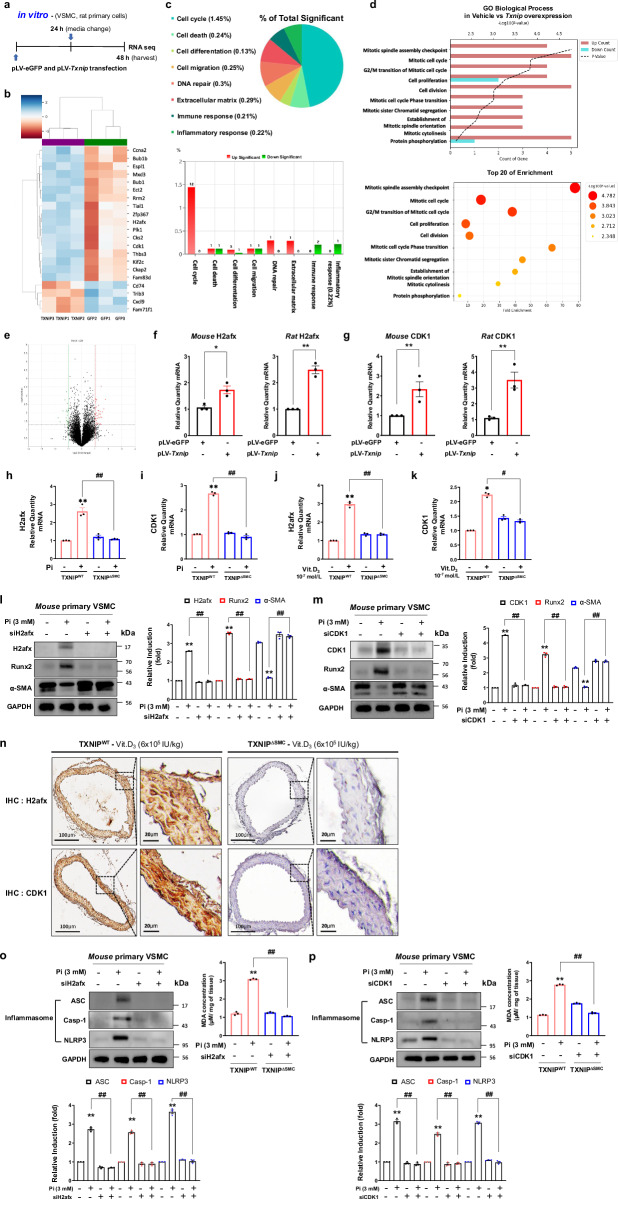
TXNIP overexpression shows changes in genes related to cell proliferation and differentiation in primary VSMCs. **a** The experimental plan for RNA sequencing of rat primary VSMCs transduced with pLV–eGFP and pLV–Txnip for 48 h. **b** A heatmap showing expression patterns of 20 genes selected among 24,000 genes. **c** Changes in expression of GO genes based on gene category chart analysis. **d** Biological processes involving 20 genes that typically increase due to TXNIP overexpression (up). The top 20 increased genes are shown as a bubble chart (down). The *x* axis represents the enrichment score of genes, and the *y* axis represents the enriched pathways of the increased genes. The color of the bubble represents the *P* value of pathway enrichment, and the size of the bubble represents the number of genes included in each pathway. **e** Volcano plot analysis for differentiation of the distribution of genes with a significant increase under TXNIP overexpression condition. **f**, **g** mRNA levels of mouse H2afx (**f**), rat H2afx (**f**), mouse CDK1 (**g**) and rat CDK1 (**g**) genes increased by TXNIP overexpression determined by quantitative RT-PCR. Results are expressed as mean ± s.d. from three independent experiments. *P < 0.05, **P < 0.01 versus pLV–eGFP (*n* = 3). **h**–**k** mRNA levels of H2afx and CDK1 genes increased by high concentration of phosphate and Vit.D3 determined by quantitative RT-PCR. Results are expressed as mean ± s.d. from three independent experiments. *P < 0.05, **P < 0.01 versis control, ^#^*P* < 0.05, ^##^*P* < 0.01 versus TXNIP^WT^ + Pi or Vit.D_3_ versus TXNIP^∆SMC^ + Pi or Vit.D_3_ (*n* = 3). The significance of differences between groups for all bar graph data was assessed using an unpaired Student’s *t*-test and ANOVA for multiple group comparisons. **l**, **m** The protein expression of H2afx, Runx2 and α-SMA was identified by immunoblotting. Protein expression band densitometry results are given as bar graphs. GAPDH expression was used as a loading control. Results are expressed as mean ± s.d. from three independent experiments. **P < 0.01 versus control, ^##^*P* < 0.01 versus Pi versus siH2afx + Pi or Pi versus siCDK1 + Pi (*n* = 3 per group). The significance of differences between groups for all bar graph data was assessed using an unpaired Student’s *t*-test and ANOVA for multiple group comparisons. **n** The expression of H2afx and CDK1 in VSMCs was assessed by IHC staining (positive staining: brown). Scale bars, 100 μm (left) and 20 μm (right). The right panels are magnifications of the areas enclosed in squares in the left panels (*n* = 3 per group). ASC, Casp-1 and NLRP3 IHC expression levels were visualized using an optical microscope. **o**, **p** The protein levels of ASC, Caspase-1 and NLRP3 were analyzed by immunoblotting in primary VSMCs transfected with siH2afx (**o**) and siCDK1 (**p**). Protein expression band densitometry results are given as bar graphs. GAPDH expression was used as a loading control. Results are expressed as mean ± s.d. from three independent experiments. **P < 0.01 versus control, ^##^*P* < 0.01 versus Pi versus siH2afx + Pi or Pi versus siCDK1 + Pi (*n* = 3 per group). The significance of differences between groups for all bar graph data was assessed using an unpaired Student’s *t*-test and ANOVA for multiple group comparisons.

To investigate the molecular mechanisms involved in TXNIP-induced calcification of VSMCs, RNA sequencing was performed 48 h after TXNIP overexpression in rat VSMCs (Fig. 8a). Heatmap analysis results showed that, among approximately 24,000 genes expressed by TXNIP overexpression (data not shown), 20 genes with differentially increased expression were identified (Fig. 8b). When gene category chart analysis was used to investigate which Gene Ontology (GO) genes typically showed what type of change in expression, the results showed that the most significant changes in expression were found in genes related to cell cycle that mediate cell proliferation, while other genes related to cell migration or differentiation were also identified (Fig. 8c). In addition, analysis of the biological processes of genes that were increased by TXNIP overexpression showed that most genes had a pattern of expression related to cell proliferation and differentiation, similar to gene category chart analysis results (Fig. 8d). Moreover, volcano plot analysis was performed to easily differentiate the distribution of significant genes due to TXNIP overexpression. Genes to the left of the green vertical line represent genes with more than a 2-fold decrease; genes to the right of the red vertical line represent genes with more than a 2-fold increase; and genes above the horizontal black line represent genes with a *P* value of ≤0.05. Genes selected through RNA sequencing showed significant differences, and the name of each gene with increased plot position was indicated (Fig. 8e). Moreover, this was confirmed by quantitative reverse transcription polymerase chain reaction (RT-PCR) analysis. Among the 20 genes that increased after TXNIP overexpression, the top two genes that showed the highest increase were selected, and differences in the mRNA level of the top two genes due to TXNIP overexpression in primary mouse and rat VSMCs were investigated. Consequently, it was determined that among the two genes exhibiting the highest upregulation in response to TXNIP overexpression, the mRNA levels of the H2afx and CDK1 genes were substantially elevated (Fig. 8f, g). To further explore the association between TXNIP expression and VSMC calcification signaling, we performed quantitative RT-PCR analyses. These analyses assessed the mRNA expression levels of the H2afx gene, identified via RNA sequencing as a mitotic cell cycle-related marker, and the CDK1 gene under conditions that induce calcification. In response to an osteogenic medium enriched with high-dose Pi and Vit.D_3_, both H2afx and CDK1 exhibited elevated mRNA levels in primary VSMCs derived from TXNIP^WT^ mice. Conversely, the upregulation of these genes was mitigated upon the deletion of TXNIP (Fig. 8h–k). These results indicate that TXNIP overexpression may facilitate VSMC calcification by enhancing cell proliferation, thereby driving phenotypic transformations and osteoblast-like differentiation in VSMCs.

To investigate the roles of CDK1 and H2afx on vascular calcification, genetic depletion was conducted with siRNA system. The immunoblotting analysis revealed that knockdown of CDK1 and H2afx inhibited the protein expression of Runx2 and significantly reversed the level of α-SMA of VSMCs, suggesting that CDK and H2afx are involved in the TXNIP-mediated vascular calcification of VSMCs (Fig. 8l, m). Consistent with in vitro system, the CDK1 and H2afx expressions markedly increased in the TXNIP^WT^ mouse aortas after the high-dose administration of Vit.D_3_, whereas their expressions decreased in the TXNIP^∆SMC^ mouse aortas (Fig. 8n). We also investigated how H2afx and CDK1 function in the oxidative stress and inflammasome activation was induced by TXNIP. The western blot results showed marked Pi-induced increases in MDA levels and protein expressions of the three components of the NLRP3 inflammasome in the primary VSMCs. The MDA levels and protein expressions of the NLRP3 inflammasome-related markers were significantly decreased by siRNAs against *H2ax* and *Cdk1*, suggesting that the TXNIP-dependent H2afx–CDK1 pathway promotes oxidative stress and inflammasome activation (Fig. 8o, p). Taken together, these results indicate that the TXNIP-dependent activation of CDK1 and H2afx is strongly associated with the development of vascular calcification.

## Discussion

This investigation unveils the pivotal role of TXNIP in vascular calcification, specifically emphasizing its modulation through targeted genetic interventions in smooth muscle cells. Our findings underscore that TXNIP acts as a crucial facilitator of oxidative stress and mitochondrial dysfunction, both of which are integral to the calcification processes within the vascular system. These results are significant as they link the molecular changes at the cellular level with large-scale pathological outcomes, providing a bridge between genetic modifications and observable disease phenotypes. Furthermore, public human GEO databases (GDS3712 and GDS3980) showed that TXNIP was increased in humans with nephrosclerosis and DM (Supplementary Fig. 1). This suggests that regulating TXNIP can ameliorate disease conditions that may cause calcification.

Intimal and medial calcifications are distinct in their roles in CVD, with different implications for disease progression and prognosis. Intimal calcification is typically associated with atherosclerosis and is found within plaques, where its impact on plaque stability is debated. Some studies suggest that small, spotty calcifications may promote plaque rupture by increasing mechanical stress on the fibrous cap^42^, while larger, more dense calcifications might stabilize the plaque^43^. By contrast, medial calcification occurs within the arterial wall’s medial layer and is more commonly linked to aging and conditions such as diabetes or CKD, leading to vascular stiffening and contributing to hypertension and left ventricular hypertrophy^3^. Regarding TXNIP, conflicting reports exist about its role in vascular calcification. Some research indicates that TXNIP promotes oxidative stress and VSMC osteogenic differentiation in vitro, thus promoting calcification^44,45^. However, another study suggested that TXNIP may have a protective role in intimal calcification by modulating the osteochondrogenic transition of VSMCs without affecting atherosclerotic region formation and lipid profile in vivo^46^. This duality highlights the complexity of vascular calcification mechanisms and the need for further investigation.

Inflammation in VSMCs is associated with atherosclerosis and other vascular inflammatory diseases that are essential for the development of vascular calcification^41^. A previous study reported that TXNIP^∆SMC^ mice showed reduced expression of inflammation-related genes in VSMCs and protection against oxidative stress in VSMCs^5^. In addition, proinflammatory molecule expression and the cell-to-cell interaction between VSMCs and macrophages were inhibited in smooth muscle cell-specific TXNIP KO, suggesting that TXNIP could be a potential target of CVD including vascular calcification^31^. By using the TXNIP^∆SMC^ murine model, where TXNIP expression is selectively abrogated in smooth muscle cells, we observed a marked attenuation in both mitochondrial dysfunction and the progression of vascular calcification after induction with high-dose Vit.D_3_ (Figs. 2 and 6). This reduction in calcification in the TXNIP-deficient model highlights the specific role of TXNIP within VSMCs in promoting pathological mineral deposition, which is often a precursor to increased vascular stiffness and reduced elasticity, common complications in CVDs.

This study also revealed that the inhibition of TXNIP could significantly impede the expression of osteogenic differentiation markers such as Runx2, osterix and BMP2, which are critical for the conversion of VSMCs to a calcifying phenotype (Figs. 2 and 3 and Supplementary Fig. 2). By preventing this transformation, TXNIP inhibition could offer a novel preventative strategy against the calcification of the vascular matrix, which is a hallmark of several metabolic and kidney diseases. Interestingly, our study also highlighted the NLRP3 inflammasome as a significant downstream target of TXNIP, connecting oxidative stress to inflammatory responses within vascular tissues. The interaction between TXNIP and the NLRP3 inflammasome suggests a complex regulatory network where TXNIP acts as a pivotal mediator of inflammatory processes that are exacerbated under conditions of oxidative stress, thus linking metabolic disturbances directly to inflammation and vascular injury.

The link between oxidative stress, inflammation and vascular calcification is critical, as it provides a potential pathway through which TXNIP contributes to the chronic inflammatory states observed in conditions such as atherosclerosis and CKD that are frequently accompanied by vascular calcification^47^. The ability to modulate this pathway through targeted TXNIP inhibition could thus serve as a key strategy in the management of vascular health, particularly in populations at high risk for cardiovascular complications. From a therapeutic perspective, the ability of TXNIP knockdown to mitigate these pathological changes offers promising avenues for intervention. The therapeutic implications of our findings are profound, suggesting that TXNIP-targeted therapies could significantly impact the clinical management of vascular calcification, particularly in patients with underlying conditions that predispose them to heightened oxidative stress and metabolic dysregulation.

According to the findings of a recent study that utilized a model of calcification induced by β-glycerophosphate, the damage to mitochondrial function occurred due to a decrease in mitochondrial DNA (mtDNA) content and cellular energy production^23^. Our study also revealed the similar results, where exposure to calcification inducers (high-dose phosphate and Vit.D_3_) reduced mitochondrial functions by decreasing intracellular mitochondrial energy production and mtDNA copy number in VSMCs of TXNIP^WT^ mice and MMP or mitochondrial oxygen consumption rate. Such findings suggest that normal VSMC function can be maintained by restoring mitochondrial function in the VSMCs of TXNIP^∆SMC^ mice, which can respond to the vascular calcification process caused by VSMC osteogenic phenotype transformation (Fig. 6). Consequently, our exploration of the mitochondrial dynamics in the context of TXNIP expression provides valuable insights into the cellular energetics underlying vascular calcification. By elucidating the effects of TXNIP on mitochondrial function and its associated oxidative stress, our study adds a crucial piece to the puzzle of how metabolic disturbances influence vascular health and disease. The observed improvements in mitochondrial respiration and ATP production in TXNIP-deficient cells align with a reduced oxidative burden, suggesting that TXNIP’s influence on mitochondrial health is a critical factor in its pathogenic profile.

TXNIP plays a role in the intracellular oxidative stress response, inducing pathological changes of blood vessels associated with cell proliferation and inflammation^48,49^. Excessive oxidative stress may trigger abnormal cell proliferation via activating transcription factors or the signal transduction pathways in cells^50^. These reports suggest that increased expression of TXNIP promotes cell proliferation, potentially contributing to abnormal vascular remodeling and associated cardiovascular complications. In regarding the role of TXNIP in regulation of cell cycle changes (Fig. 8), TXNIP may strongly impact not only the osteogenic differentiation but also the phenotypic change of VSMCs, which is a potential therapeutic target for treating vascular diseases^51,52^. As proliferation of VSMCs is a key event in the synthetic phenotypic changes, TXNIP-mediated mitotic gene induction may contribute to the progression of the synthetic phenotypic changes. The precise role of TXNIP in phenotypic changes of VSMCs remains to be determined.

Calcification is also associated with abnormal mitotic cell cycle. Normal cell growth takes place according to the cell cycle, and the cell cycle follows a sequence by receiving signals from internal or external mechanisms^53,54^. It has been suggested that an abnormal cell cycle can cause acquired mitochondrial dysfunction, leading to hyperproliferative diseases, such as cancer and pulmonary arterial hypertension^55,56^, while also affecting vascular remodeling^41^. In our study, RNA sequencing was used to identify various genes related to the cell cycle with TXNIP overexpression, and the results showed that each gene was associated with the mitotic cell cycle (Figs. 7 and 8). Moreover, among the genes identified by RNA sequencing, CDK1 and H2afx genes showed a significant increase in primary VSMCs with high-dose Pi and Vit.D_3_ or TXNIP overexpression (Fig. 8h–k). Genetic depletion analysis using siRNA indicates that mitotic regulators are new downstream targets of TXNIP and promote vascular calcification (Fig. 8l, m). Although the RNA sequencing data did not reveal direct mechanism linking inflammation or mitochondrial dysfunction regulated by TXNIP to the increased expression of H2afx and CDK1, we found that TXNIP overexpression upregulated some genes involved in inflammation and mitochondrial dysfunction. For instance, there may be potential interactions between TXNIP and DNA polymerase subunit gamma (POLG) associated with oxidative stress and mitochondrial function. Previous reports have indicated early aging from the accumulation of mtDNA mutation in POLG mutant mouse models^57^, while another study reported that mice with POLG mutation showed accumulation of mtDNA mutation that caused increased oxidative stress and apoptosis, which promoted inflammatory responses and aging^58^. In addition, our RNA sequencing data showed increases in CXCL1, CXCL2 and CCL20 genes when TXNIP was overexpressed, although the differences were not significant. Consistent with our results, a previous study reported that TXNIP-overexpressing human astrocytes showed a significant increase in inflammatory genes such as CXCL1, CXCL2 and CCL20^59^. These observations suggest that TXNIP is a key regulator in oxidative stress and inflammation during the vascular calcification process.

It can be speculated that inflammation or oxidative stress caused by TXNIP overexpression causes the abnormal activation of intrinsic signaling molecules that regulate cell proliferation and division, promoting an abnormal cell cycle. Consequently, mitochondria proliferation, replication and division were also affected by abnormal cell cycle^56,60^, causing functional impairment with decreased mitochondrial copy number and vascular pathologies induced by inflammation and oxidative stress. However, additional studies are needed to fully understand such mechanisms, as well as to demonstrate the mechanisms by which CDK1 and H2afx genes regulate vascular calcification.

In conclusion, our study not only clarifies the role of TXNIP in vascular calcification but also positions it as a potential target for therapeutic strategies aimed at mitigating vascular stiffness and improving outcomes in patients with CVDs linked to enhanced oxidative stress and metabolic disturbances. Moving forward, it will be essential to explore the full clinical implications of TXNIP modulation in vascular health and to develop targeted therapies that could inhibit or reverse the calcification process. The integration of genetic, cellular and molecular data from our study provides a solid foundation for future therapeutic developments aimed at controlling or preventing vascular calcification through modulation of TXNIP.

## Supplementary information

Supplementary Information
