# Supplementary material for: Smooth muscle cell-specific deletion of TXNIP ameliorates medial vascular calcification
Source: Exp Mol Med. 2025 Jul 3;57(7):1519–35. doi: 10.1038/s12276-025-01474-5 (PMC12322165; doi:10.1038/s12276-025-01474-5)
Supplement: Supplementary file 1 — Supplementary Information [file 12276_2025_1474_MOESM1_ESM.pdf]

Smooth muscle cell-specific deletion of TXNIP ameliorates medial vascular calcification

Ae-Rang Hwang[1], Chang-Hoon Woo[1*]

[1]*Department of Pharmacology and Senotherapy-based Metabolic Disease Control Research Center, Yeungnam University College of Medicine, Daegu 42415, Republic of Korea,*

Supplemental materials and methods

Chemical reagents and antibodies

Alizarin red S staining reagents were purchased from Bio Basic Inc. (Markham ON, Canada). Cholecalciferol and von Kossa staining reagents (Silver nitrate) were from Sigma-Aldrich (St. Louis, MO, USA), and Monosoium were from Oriental chemical industries (OCI, Seoul, Republic of Korea) and phosphate and Disodium phosphate were from DUKSAN REAGENTS (DUKSAN, Ansan, Republic of Korea). QuantiChrom™ calcium asaay kit and QuantiChrom™ alkaline phosphatase assay kit were from Bioassay Systems (Hayward, CA, USA). Immunohistochemistry (IHC) kit were purchased from Abcam (Cambridge, MA, USA). MitoSOX™ Red, JC-1 staining reagents were purchased from Invitrogen (Carlsbad, CA, USA) and MitoTracker[R] Green were purchased Cell Signaling Technology (Danvers, MA, USA). ATP Detection Assay Kit were from Cayman Chemical (Michigan, MI, USA). Antibodies in this study were as follows: mouse anti-α-SMA (cat. no. SC-58669), mouse anti-ASC (cat. no. SC-514414) were supplied by Santa Cruz Biotechnology (Delaware, CA, USA), and mouse anti-TXNIP (cat. no. K0205-3) was from MEDICAL & BIOLOGICAL LABORATORIES (MBL) (MBL, Tokyo, JAPAN), and mouse anti-Runx2 (cat. no. #8486) was from Cell Signaling Technology (Danvers, MA, USA). Mouse anti-Caspase-1(cat. no. 22915-1-AP) was from Proteintech (Rosemont, IL, USA), and mouse anti-NLRP3(cat. no. #PA5-79740) was from Invitrogen (Carlsbad, CA, USA). Mouse anti-tubulin (cat. no. T5168) was from Sigma-Aldrich (St. Louis, MO, USA), and goat anti- mouse IgG-HRP (cat. no. GTX213111-01) and goat anti-rabbit IgG-HRP (cat. no. GTX213110-01) were from GeneTex (Irvine, CA, USA).

Western blotting analysis

Cells were exposed to radioimmunoprecipitation assay (RIPA) lysis buffer containing 0.01 mM protease inhibitor cocktail (PIC) and 1 mM phenylmethylsulfonyl fluoride (PMSF), incubated on ice for 15 min, followed by centrifugation at 15,000 rpm for 15 min. The resulted lysate was measured for protein concentration using the Bradford assay. These samples were separated by SDS-PAGE, and the proteins were transferred to membranes where antigen-antibody reaction was detected using chemiluminescence. Membranes were blocked with 5% skim milk at room temperature for 1 hr to prevent non-specific binding, incubated overnight with primary antibodies (1:1000), then with secondary antibodies (1:5000 at room temperature for 1 hr) to detect desired proteins. Western blot bands were quantified using housekeeping genes, such as GAPDH and tubulin.

Quantitative Real Time RT-PCR

RNA was extracted using TRIzol reagent (Invitrogen, Carlsbad, CA, USA) and mRNA levels were assessed by RT-PCR (qRT-PCR). Data analysis was conducted using ABI PRISM 7500 (Applied Biosystems) with 1 µg of template cDNA and power SYBR green PCR master mix (Applied Biosystems). Quantification was performed using corrected ΔΔCt method. The primers used to amplify DNA sequences were as follows: mouse α-SMA: forward, 5′-TTTCCAAATCATTCCTGCCC-3′ and reverse, 5′- CGCTCTCAAATACCCCGTTT-3′; mouse Runx2: forward, 5′-AGATGATGACACTGCCACCTCTG-3′ and reverse, 5′-GCTCTCAGTGAGGGATGAAATGC-3′; mouse Osterix: forward, 5′-GAAAGGAGGCACAAAGAAG-3′ and reverse, 5′-CACCAAGGAGTAGGTGTGTT-3′; mouse BMP2: forward, 5′-TTTGCACCAAGATGAACACAGC-3′ and reverse, 5′-GCTTCCGCTGTTTGTGTTTG-3′; mouse ASC: forward, 5′-CTCTGTATGGCAATGTGCTGAC-3′ and reverse, 5′- GAACAAGTTCTTGCAGGTCAG-3′; mouse Caspase-1: 5′- GAGCTGATGTTGACCTCAGAG-3′ and reverse, 5′-CTGTCAGAAGTCTTGTGCTCTG-3′; mouse NLRP3: forward, 5′-GTGGAGATCCTAGGTTTCTCTG-3′ and reverse, 5′- CAGGATCTCATTCTCTTGGATC-3′; mouse H2afx: forward, 5′- AACGACGAGGAGCTCAACAAGC-3′ and reverse, 5′-TGGCGCTGCTCTTCTTGGGCA-3′; rat H2afx: forward, 5′- GGGCCTAGCTATCCCTCTCCCT-3′ and reverse, 5′- CTGCAAAAGTTCCAGTTCAGAAGCCAGA-3′; mouse CDK1 5′-GCTTTTCCACGGCGACTCAG-3′ and reverse, 5′- ATCCAAGCCGTTCTCGTCCA-3′; rat CDK1: forward, 5′- TCTTCGCTCGTTAAGAGTTAC-3′ and reverse, 5′- ATCTGCCAGTTTGATTGTTC-3′; mouse GAPDH: forward, 5′-ACAAGATGGTGAAGGTCGGT-3′ and reverse, 5′-AGCTTCCCATTCTCAGCCTTGA-3′; mouse Cox-1: forward, 5′- CTGAGCGGGAATAGTGGGTA-3′ and reverse, 5′- TGGGGTCCGATTATTAGTG-3′; mouse b-globin: forward, 5′-GCACCTGACTGATGCTGAGAA-3′ and reverse, 5′- TTCATCGGCGTTCACCTTTCC-3′.

Alizarin red S staining

Alizarin red S staining was used to detect calcification of VSMCs according to the following protocol: calcified VSMCs were washed three times with 1× PBS and exposed to 20% Alizarin red S solution (Sigma, pH 4.2) at room temperature for 20 min. Tissues were also dehydrated and fixed in paraffin for pathological analysis and immunohistochemistry, then sectioned into 4 μm slices for microscopic examination. The sections were deparaffinized with xylene twice for 2 min each, rehydrated with 100% ethanol twice for 2 min each, 95% ethanol for 1 min, 90% ethanol for 1 min, 80% ethanol for 1 min, 70% ethanol for 1 min, and washed with running tap water for 5 min. Afterwards, the sections were stained and calcium accumulation in VSMCs were observed using an optical confocal microscope (Leica, Bannockburn, IL, USA).

For von Kossa staining

For von Kossa staining, the calcified VSMCs were washed with 1X PBS 3 times and then cells were exposed to 5% silver nitrate solution (Sigma–Aldrich, St. Louis, MO, USA) placed under UV light for 60 min or longer (60–100 watt light bulb or strong sunlight can be used to replace UV light if UV light source is not available). The sections were deparaffinized with xylene twice for 2 min each, rehydrated with 100% ethanol twice for 2 min each, 95% ethanol for 1 min, 90% ethanol for 1 min, 80% ethanol for 1 min, 70% ethanol for 1 min, and washed with running tap water for 5 min. Afterwards, the tissues were treated with 5% silver nitrate solution to observe calcium accumulation using an optical confocal microscope.

Immunohistochemistry

Aorta tissues were fixed in 10% buffered formalin, embedded in paraffin and sectioned into 3 μm slices for immunohistochemistry. The calcified aorta sections were all equally deparaffinized and exposed to primary antibodies for mouse anti-ASC (1:200), caspase-1 (1:100), and NLRP3 (1:200) overnight at 4°C and attached with secondary antibodies the next day to visualize each target protein. All procedures were conducted using the HRP/DAP (ABC) detection IHC kit (Abcam, Cambridge, UK) following the manufacturer’s instructions. Each protein expression in calcified aorta tissues was observed using an optical confocal microscope.

Calcium deposition and ALP activity assay

Blood concentrations of calcium deposition (DICA–500) and ALP activity (DALP–250) were measured using commercially available kits QuantiChrom calcium assay kit and alkaline phosphatase assay kit (Hayward, CA, USA) as described by manufacture's protocol.

Aortic Calcification by OsteoSense 680EX

At 24 hr before sacrifice, mice were injected with 0.2 μM OsteoSense 680EX (NEV10020EX; Perkin Elmer, MA, USA). After euthanasia, cardiovascular system from mice was perfused with saline, and the aorta were collected. And then, analysis on a black surface to abrogate fluorescence reflectance. The microcalcifications nodules were then observed with a Spectral Ami/Ami X (Tucson, AZ, USA).

Transient transfection of small interfering RNA (siRNA)

For TXNIP silencing, cells were transiently transfected with 100 pmol/mL of specific mouse and rat siRNA against *Txnip* by Solfect (Daegu, Republic of Korea) following protocols provided by manufacturer. The targeting mouse and rat siTxnip (VDUP1 siRNA, sc–44944 and sc–270490) was obtained from Santa Cruz (Delaware, CA, USA). A non-specific control siRNA from Bioneer (Daejeon, Republic of Korea) was used as a negative. Transient transfection of siRNA was performed at 70–80% confluence of mouse and rat vascular smooth muscle primary cell. Simultaneously with siRNA transfection, 3 mM Pi was treated every other day for 7 days, and VSMC calcification was detected using protein quantification or Alizarin Red S staining.

TXNIP overexpression using lentivirus system

To generate lentivirus particles encoding mouse *Txnip*, HEK293T cells were cotransfected with psPAX2 (#12260), pMD2.G (#12259), pLV-eGFP (#36083), (Addgene, Cambridge, MA, USA) or pLV-Txnip (Self-exclusion). After 48 hr lentivirus particles were obtained using concentration kit with cultured medium, and followed by titration. To overexpression the TXNIP, VSMCs were transduced with lentivirus particles for 48 hr and applied for further experiments.

Oxidative status analysis using MDA

Oxidation stress marker levels of malondialdehyde (MDA) were detected in aorta tissue and primary cell using the ELISA and Western blot method. After being carefully washed with PBS, tissues and cells were lysed with 250 μl RIPA buffer, homogenized, and centrifuged at 1,600g for 10 min at 4°C. Afterwards, the supernatant was carefully collected and incubated at room temperature for at least 10 min to a maximum of 30 min until it became clear. Samples were stored at -80°C if not analyzed immediately. Next, 150 μl of the supernatants were transferred to a 96-well plate and analyzed using a microplate reader at 530–540 nm, following the instructions provided with the kit.

Mitochondria isolation

For mitochondrial isolation, aorta or fresh cells were homogenized in buffer A containing 100 mM KCl, 50 mM Tris base, 5 mM $MgCl_2.6H_2O$, 1.8 mM ATP, and 1 mM EDTA as well as protease, then the sample was transferred and was by centrifuged at 720*g* (4˚C) for 5 min.

Measurement of mitochondrial respiratory capacity

Mitochondria were isolated from rat vascular smooth muscle cell samples and mitochondrial oxygen consumption was analyzed using an Oxytherm System (Hansatech Instruments, Pentney, UK), as previously described [1]. Briefly, fresh tissues were homogenized in buffer A containing 1.8 mM ATP, 1 mM EDTA, 100 mM KCl, 50 mM Tris base, 5 mM $MgCl_2 \cdot 6H_2O$ and a protease, and further centrifuged at 720*g* at 4˚C for 5 min. The supernatant was transferred to a fresh tube and centrifuged at 1000*g* for 5 min, and the resulting supernatant was discarded. The precipitated mitochondria were resuspended in buffer A and further centrifuged at 9000*g* for 5 min. After discarding the supernatant, the mitochondrial pellets were resuspended in respiratory buffer (2 mM $MgCl_2$, 20 mM HEPES, 2.5 mM KH_2PO_4, and 125 mM KCl, pH adjusted to 7.2). The isolated mitochondria were added to the chamber of an Oxytherm machine along with 950 µl of respiratory buffer and warmed to 37˚C under constant agitation. Mitochondrial respiration was assessed in the absence of substrate (State 1), in the presence of 5 mM pyruvate/2.5 mM malate or 5 mM palmitoyl-carnitine/2.5 mM malate (State 2), and in the presence of 2.5 mM adenosine diphosphate (ADP) (State 3). ATP synthase activity was inhibited by the addition of 2.5 µM oligomycin, leading to State 4 respiration. Oxygen consumption rates were normalized to the concentration of isolated mitochondria and were expressed as nanomoles/mL/µg. The respiratory control ratio (RCR), which represents mitochondrial coupling efficiency, was calculated as the ratio of state 3 to state 4 respiration.

Mitochondrial oxygen consumption rate

The OCR of VSMCs was measured using the Seahorse Extracellular Flux (XF) analyzer (Agilent Cell Analysis technology, Santa Clara, CA, USA). VSMCs were seeded at 1×104 cells per well, and 200 µL of XF Calibrant solution was added to each well of a Seahorse XFe96 plate 1 day before the OCR analysis for overnight incubation at 37°C in a non-CO2 incubator. During the analysis, 1.5 µM oligomycin, 1 µM FCCP, and 0.5 µM rotenone/antimycin A were injected at indicated times to assess changes in cellular respiration. OCR data were acquired per the manufacturer's protocol.

Assessment of ROS analysis using MitoSOX™

Mitochondrial superoxide was measured in live VSMCs using MitoSOX™ Red and MitoTracker® Green. Calcified VSMCs were washed three times with 1× PBS, exchanged with serum-free DMEM 5

μmol/L MitoSOX™ Red and 400 nmol/L MitoTracker® Green, and incubated in the dark at 37 °C with 95% air and 5% CO2 for 10 min. Lastly, the cells were washed three times with 1× PBS, and mitochondrial superoxide was detected using a fluorescence microscope at 561 nm and 488 nm.

ATP measurements

ATP can be measured using an enzyme called luciferase, which catalyzes the oxidation of luciferin in the presence of ATP and produces light as a result. First, media was removed from the cultured plate and washed three times with cold 1× PBS. Afterwards, the cells were lysed with 1× ATP assay sample buffer (100 μl per well), scraped with a scraper, and collected. The lysate was incubated on ice for 10 min. Cells were Homogenized the cells by pipetting the up and down several time. Cells were homogenized and then centrifuged at 13,000g for 5 min at 4˚C. The resulting supernatant and assayed for ATP with an ATP detection assay kit (#700410) – Luminescence from Cayman (Michigan, MI, USA) to measure the luminescence.

Mitochondrial DNA copy number

Mitochondrial DNA (mtDNA) copy number was analyzed using real-time qPCR with power SYBR green PCR master mix (Applied Biosystems) and 1 μg of template cDNA, according to previously described qPCR assay protocols. In addition, quantification was performed and corrected using the ΔΔCt method [2].

JC-1 mitochondrial potential

The mitochondrial potential was assessed using the JC-1 dye. VSMCs were exposed to serum-free DMEM containing JC-1 dye (20 nM), incubated in the dark at 37°C for 15 min. Afterwards, the media was removed and cells were washed three times with 1× PBS, then applied for JC-1 dye fluorescence intensity under a fluorescence microscope. Mitochondrial membrane potential was estimated based on the ratio of J-aggregate (540 nm excitation and 590 nm emission) to JC–1 monomer (490 nm excitation and 540 nm emission) fluorescence intensities. For flow cytometry assay with JC-1, VSMCs exposed to JC-1 dye (20 nM) were washed three times with 1× PBS before being treated with trypsin and collected, then centrifuged at 3,000 rpm. The obtained pellets were resuspended in 1× PBS (500 μl) and JC-1 dye fluorescence intensity was assessed using flow cytometry analysis.

QuantSeq 3' mRNA-Sequencing

Total RNA was isolated using Trizol reagent (Invitrogen). RNA quality was assessed by Agilent TapeStation 4000 system (Agilent Technologies, Amstelveen, The Netherlands), and RNA quantification was performed using ND-2000 Spectrophotometer (Thermo Inc., DE, USA).

RNA_seq analyses were performed using the Quant Seq 3' mRNA–seq library prep kit from Lexogen GmbH (Lexogen, Wien, Austria) to identify differentially expressed genes in the vascular smooth muscle cells of the $TXNIP^{WT}$ mice, as previously described. In brief, each total RNA was prepared and an oligo–dT primer containing an Illumina–compatible sequence at its 5' end was hybridized to the RNA and reverse transcription was performed. After degradation of the RNA template, second strand synthesis was initiated by a random primer containing an Illumina compatible linker sequence at its 5' end. The double–stranded library was purified by using magnetic beads to remove all reaction components. The library was amplified to add the complete adapter sequences required for cluster generation. The finished library is purified from PCR components. High–throughput sequencing was performed as single–end 75 sequencing using Next Seq 550 (Illumina, CA, USA). Data analysis was performed as follows: A quality control of raw sequencing data was performed using FastQC [3]. Sequenced reads were trimmed for adapter sequences and low-quality filtered using bbduk. Then the clean reads were mapped to the reference genome using Bowtie2 [4]. The quantification of reads was processed using Bedtools [5]. The Read Counts were processed based on TMM+CPM normalization method using EdgeR [6]. Data mining and graphic visualization were performed using ExDEGA (Ebiogen Inc., Korea).

In silico analysis of relative gene expression

The TXNIP expression profiling data sets are available from the National Center for Biotechnology Information Gene Expression Omnibus (GEO) database [http://www.ncbi.nlm.nih.gov/geo/, accession numbers: GDS3712 (control vs. Nephrosclerosis patients) and GDS3980 (control vs. Type 2 diabetes patients)]. The relative expression levels of TXNIP in the experimental group of each data set were analyzed in comparison with the control values. Visualization of TXNIP gene expression was carried out in GraphPad Prism.

Supplementary figure and legends

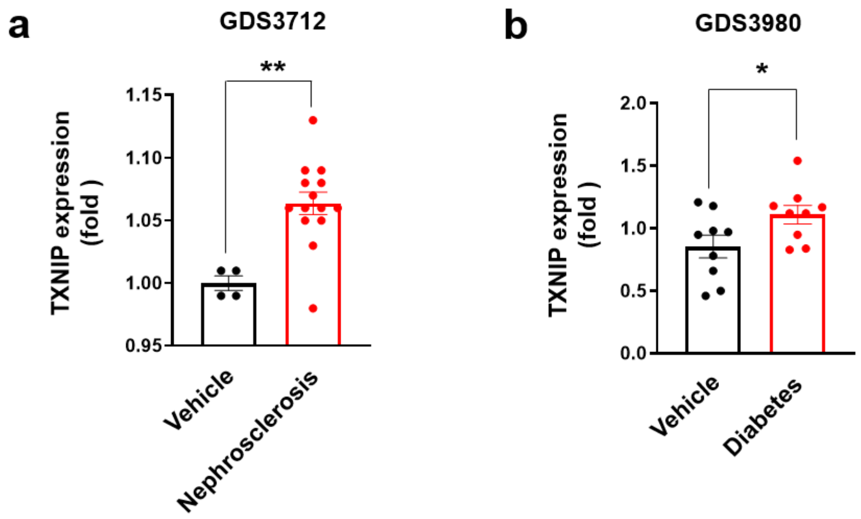

Supplementary Fig 1. Increased TXNIP expression in human diseases.

(a and b) GEO databases [GDS3712 (vehicle, n=4 / nephrosclerosis, n=12) and GDS3980 (vehicle, n=9 / diabetes, n=9)] were used to analyze TXNIP mRNA levels expressed differentially in subjects with nephrosclerosis and DM. Data are displayed as the mean ± SE. Statistical analyses between two groups were performed using an unpaired Student's *t*-test. *$p < 0.05$, **$p < 0.01$ vs. GEO control. The significance of differences between groups for the bar graph data was assessed using an unpaired Student's t-test.

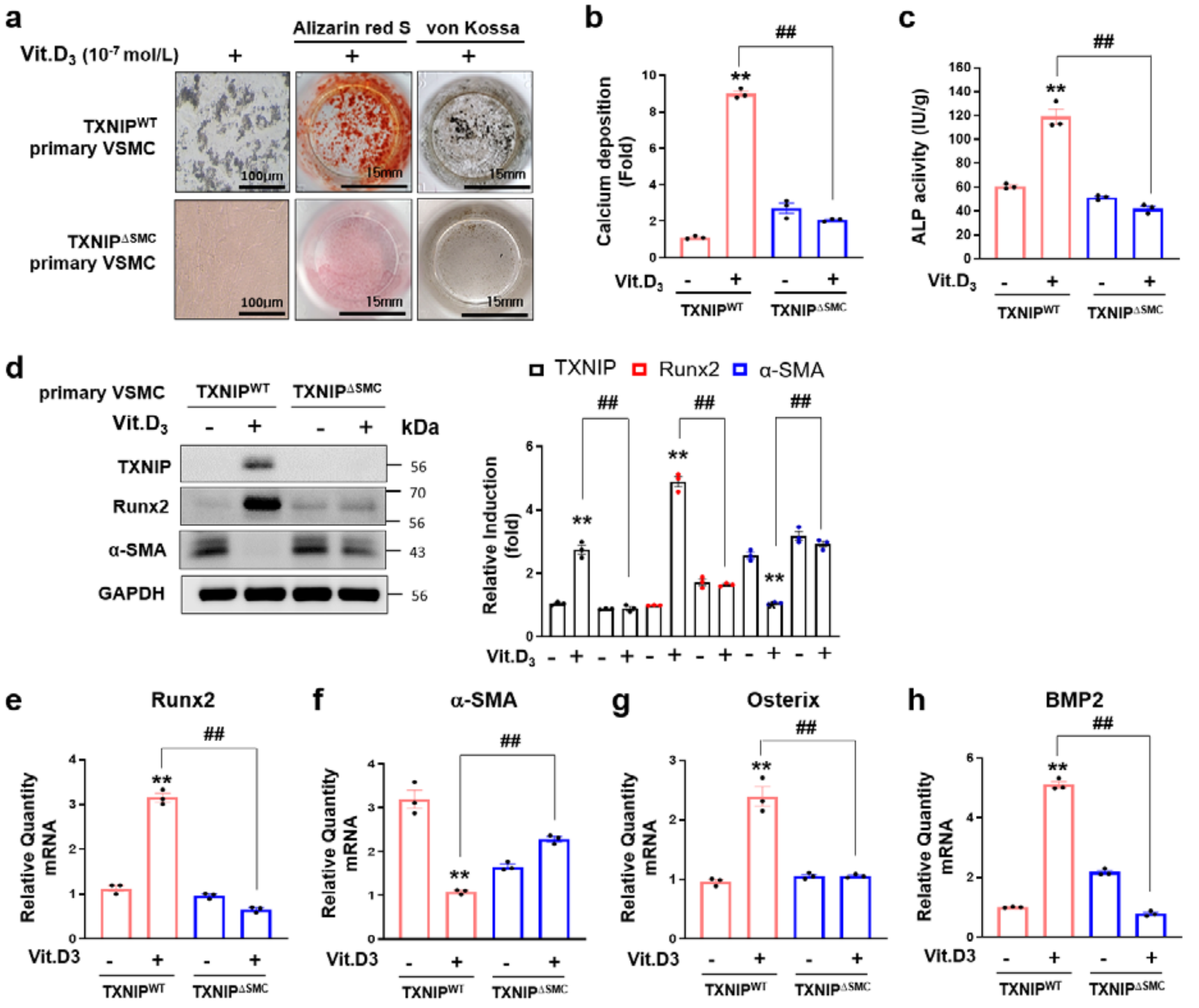

Supplementary Fig 2. High–dose Vit.D$_3$ increases calcification in a TXNIP–dependent manner in mouse primary vascular smooth muscle cells.

Primary VSMCs isolated from TXNIP^{WT} and TXNIP$^{\Delta SMC}$ mice were cultured in a medium containing DMEM or high–dose Vit.D$_3$ (10^{-7} mol/L) replaced in 2-day intervals and cultured in an osteogenic medium containing high–dose Vit.D$_3$ for 14 days. (a) Calcification due to micro-Calcium accumulation was visualized by Alizarin red S (positive staining: red) or von Kossa staining (positive staining: black) Scale bar = 100 µm and well diameter = 15 mm (n=3 per group). (b and c) Analysis of Calcium content and ALP activity in primary VSMCs of TXNIP^{WT} and TXNIP$^{\Delta SMC}$ mice after exposure to Vit.D$_3$. Results are expressed as mean ± SD from three independent experiments. ** $p < 0.01$ vs. control, ## $p < 0.01$ versus. TXNIP^{WT} + Vit.D$_3$ vs. TXNIP$^{\Delta SMC}$ + Vit.D$_3$ (n=3 per group). (d) Protein expressions of TXNIP, Runx2, and α–SMA in primary VSMCs of TXNIP^{WT} and TXNIP$^{\Delta SMC}$ mice were analyzed by immunoblotting. Protein expression band densitometry results are given as bar graphs. GAPDH antibodies were used as loading control. **$p < 0.01$ vs. control, ##$p < 0.01$ versus. TXNIP^{WT} + Vit.D$_3$ vs. TXNIP$^{\Delta SMC}$ + Vit.D$_3$ (n=3 per group). (e–h) The transcription levels of calcification-related genes

(Runx2, Osterix, and BMP2) and VSMC contractile gene marker (α–SMA) were analyzed by qPCR. Results are expressed as mean ± SD from three independent experiments. $^{**}p < 0.01$ vs. control, $^{\#\#}p <$ 0.01 versus. $TXNIP^{WT}$ + Vit.D_3 vs. $TXNIP^{\Delta SMC}$ + Vit.D_3 (n=3 per group). The significance of differences between groups for all bar graph data was assessed using an unpaired Student's t-test and analysis of variance (ANOVA) for multiple group comparisons.

Supplementary References

1. Abedin, M., Tintut Y. & Demer L. L. Vascular calcification: mechanisms and clinical ramifications. *Arterioscler Thromb Vasc Biol* **24**, 1161-1170 (2004).
2. Johnson, R. C., Leopold J. A. & Loscalzo J. Vascular calcification: pathobiological mechanisms and clinical implications. *Circ Res* **99**, 1044-1059 (2006).
3. de Sena Brandine, G. & Smith A. D. Falco: high-speed FastQC emulation for quality control of sequencing data. *F1000Res* **8**, 1874 (2019).
4. Langmead, B. & Salzberg S. L. Fast gapped-read alignment with Bowtie 2. *Nat Methods* **9**, 357-359 (2012).
5. Quinlan, A. R. & Hall I. M. BEDTools: a flexible suite of utilities for comparing genomic features. *Bioinformatics* **26**, 841-842 (2010).
6. Robinson, M. D., McCarthy D. J. & Smyth G. K. edgeR: a Bioconductor package for differential expression analysis of digital gene expression data. *Bioinformatics* **26**, 139-140 (2010).
